# Prognostic mutational subtyping in de novo diffuse large B-cell lymphoma

**DOI:** 10.1186/s12885-022-09237-5

**Published:** 2022-03-03

**Authors:** Eugene Kim, Yanwen Jiang, Tao Xu, Alexandra Bazeos, Andrea Knapp, Christopher R. Bolen, Kathryn Humphrey, Tina G. Nielsen, Elicia Penuel, Joseph N. Paulson

**Affiliations:** 1 Department of Biostatistics, Product Development, Genentech, Inc, 1 DNA Way, MS 444A, South San Francisco, CA 94080 USA; 2 Oncology Biomarker Development, Genentech, Inc, South San Francisco, CA USA; 3 PHC Analytics, Product Development, F. Hoffmann La-Roche Ltd, Basel, Switzerland; 4 Roche Products Ltd, Welwyn Garden City, England; 5 Pharma Development Clinical Oncology, F. Hoffmann La-Roche Ltd, Basel, Switzerland; 6 Bioinformatics & Computational Biology, Genentech, Inc, South San Francisco, CA USA

**Keywords:** Diffuse large B-cell lymphoma, Obinutuzumab, Genomics, Next-generation sequencing, Rituximab, Venetoclax, Real world

## Abstract

**Background:**

Diffuse large B-cell lymphoma (DLBCL) is a heterogeneous disease defined using a number of well-established molecular subsets. Application of non-negative matrix factorization (NMF) to whole exome sequence data has previously been used to identify six distinct molecular clusters in DLBCL with potential clinical relevance. In this study, we applied NMF-clustering to targeted sequencing data utilizing the FoundationOne Heme® panel from the Phase III GOYA (NCT01287741) and Phase Ib/II CAVALLI studies (NCT02055820) in *de novo* DLBCL. Biopsy samples, survival outcomes, RNA-Seq and targeted exome-sequencing data were available for 423 patients in GOYA (obinutuzumab [G]-cyclophosphamide, doxorubicin, vincristine, and prednisone [CHOP] vs rituximab [R]-CHOP) and 86 patients in CAVALLI (venetoclax+[G/R]-CHOP).

**Results:**

When the NMF algorithm was applied to samples from the GOYA study analyzed using a comprehensive genomic profiling platform, four of the six groups previously reported were observed: *MYD88/CD79B*, *BCL2/EZH2*, *NOTCH2/TNFAIP3*, and no mutations. Mutation profiles, cell-of-origin subset distributions and clinical associations of *MYD88/CD79B* and *BCL2/EZH2* groups were similar to those described in previous NMF studies. In contrast, application of NMF to the CAVALLI study yielded only three; *MYD88/CD79B*-, *BCL2/EZH2*-like clusters, and a no mutations group, and there was a trend towards improved outcomes for *BCL2/EZH2* over *MYD88/CD79B*.

**Conclusions:**

This analysis supports the utility of NMF used in conjunction with targeted sequencing platforms for identifying patients with different prognostic subsets. The observed trend for improved overall survival in the *BCL2/EZH2* group is consistent with the mechanism of action of venetoclax, suggesting that targeting sequencing and NMF has potential for identifying patients who are more likely to gain benefit from venetoclax therapy.

**Supplementary Information:**

The online version contains supplementary material available at 10.1186/s12885-022-09237-5.

## Significance statement

Molecularly defined DLBCL subgroups provide additional context for treatment decisions in addition to classical prognostic features (IPI, BCL2/MYC status).

## Introduction

Diffuse large B-cell lymphoma (DLBCL) is a genetically and clinically heterogeneous disease [1, 2]. Since 2000, gene expression profiling has been used to categorize DLBCL into distinct cell-of-origin (COO) subtypes, which include activated B-cell-like (ABC) and germinal center B-cell-like (GCB) subtypes [3–5]. These subtypes arise from different stages of normal B-cell development and their prognostic impact has been confirmed in several studies. Notably, in retrospective analyses of patients with DLBCL receiving rituximab (R) plus cyclophosphamide, doxorubicin, vincristine, and prednisone (CHOP; R-CHOP), the ABC subtype has been associated with less favourable outcomes compared with the GCB subtype [6, 7]. However, the prognostic difference based on COO alone has not always translated to clinical observations in the R-CHOP era [8–10]. Clinically relevant genetic subgroups that contribute to the molecular profile of the COO subtypes have been described and may yield greater discrimination and stratification than COO alone (e.g. *CD79b* or *MYD88* mutations in ABC and *BCL2* translocation in GCB) [11–15].

With advances in next-generation sequencing, the ability to further interrogate genetic drivers to refine prognostic subgroups over and above COO subtypes has generated considerable interest. Using whole-exome and transcriptome sequencing of tumors from a cohort of 1001 newly diagnosed patients with DLBCL, Reddy et al developed a prognostic model comprising genetic alterations, COO DLBCL subtype, International Prognostic Index (IPI) score and dual *MYC* and *BCL2* expression, that outperformed molecular or clinical factors alone (COO, *MYC/BCL2* expression, IPI) in terms of prognostic ability for overall survival (OS) [16]. Further elucidation of some of the clinical and genetic heterogeneity in transcriptionally defined COO subsets from *de novo* DLBCL identified several common mutational profiles with distinct prognostic impact [2, 17]. Applying an unsupervised prognostic clustering approach, non-negative matrix factorization (NMF), Chapuy and colleagues were able to identify six distinct molecular clusters with potential clinical relevance leveraging whole exome sequencing; these included two distinct subsets of ABC (one enriched for mutations in *MYD88* and *CD79B*, and another for *BCL6* and *NOTCH* mutations) and a GCB subset enriched for *BCL2* translocations and mutations in *CREBBP* and *EZH2.* These analyses demonstrated the value for incorporating multiple data types to identify prognostic subsets. As an additional step Wright et al. [18] developed a probabilistic algorithm, LymphoGen from multi-platform analyses to delineate clinically actionable subsets. These instrumental advances expand DLBCL subsets by including genetic data but rely on complex datasets/analyses from WES. FMI targeting panels have potential to make this type of analysis more accessible and therefore more actionable in the clinical setting.

In the current study, we applied the NMF-clustering approach on targeted exome-sequencing data from the Phase III GOYA study (NCT01287741)^5^ and the Phase Ib/II CAVALLI study (NCT02055820) [19, 20] to scrutinize known molecular clusters and novel prognostic groups in *de novo* DLBCL. The randomized GOYA study evaluated R-CHOP or obinutuzumab (G)-CHOP in patients with previously untreated advanced-stage DLBCL. Results from the final analysis of GOYA reported no significant difference in progression-free survival (PFS) in patients treated with G-CHOP vs R-CHOP [5], and hence the treatment arms were pooled for this secondary analysis. In the subsequent CAVALLI study, addition of the highly selective BCL2 inhibitor venetoclax to R-CHOP was associated with an improvement in PFS and OS in patients with previously untreated BCL2 positive (by immunohistochemistry [IHC]) DLBCL, when compared with a matched historical control group treated with R-CHOP alone in GOYA [19, 20]. Inclusion of data from the CAVALLI study in our genomic analysis enabled us to explore whether outcomes are improved for patients with *BCL2*-rich NMF-clustering characteristics (i.e. the Chapuy defined GCB subset that is enriched for BCL2 translocations) who are treated with venetoclax. We also evaluated a real-world DLBCL cohort from a de-identified clinico-genomic database to determine whether the clusters identified in the GOYA study could be replicated in a real-world cohort. Our aim was to demonstrate that molecular clusters of DLBCL can be identified using genomic information obtained from a clinically validated next-generation targeted sequencing platform and potentially inform treatment decisions.

## Methods

### GOYA and CAVALLI study design and participants

The design for the GOYA (NCT01287741) and CAVALLI trials (NCT02055820) has previously been described [5, 19]. Both GOYA and CAVALLI included patients with previously untreated CD20-positive DLBCL. Inclusion criteria for both studies are briefly described (Supplementary Methods, online only). In GOYA, patients were treated with eight 21-day cycles of G 1000 mg (Days 1, 8 and 15, Cycle 1; Day 1, Cycles 2-8) or R 375 mg/m^2^ (Day 1, Cycles 2-8) plus 6-8 cycles of CHOP. In CAVALLI, patients received eight 21-day cycles of venetoclax 800 mg (Days 1-4, Cycle 1; Days 1-10, Cycles 2-8) plus R or G (Cycles 1-8) and CHOP (Cycles 6-8).

Using patient-derived biopsy samples, clinical outcomes data (OS and PFS), the FoundationOne® Heme (F1H) assay (Foundation Medicine Inc. [FMI], Cambridge, MA, USA), and high-throughput transcriptome sequencing, data were available (biomarker-evaluable population) for 423 patients from the GOYA trial (intent-to-treat [ITT] population, *n* = 1418; data cut-off January 31, 2018; median follow-up 3.9 years) and for 86 patients from the CAVALLI trial (ITT, *n* = 267, *n* = 206 evaluable for efficacy and safety, venetoclax +[G/R]-CHOP, data cut-off June 28, 2019, median follow-up 32.2 months).

Genomic alterations were identified via comprehensive genomic profiling of >300 cancer-related genes on FMI's next-generation sequencing-based FoundationOne® panel [21].

At the time of the analysis, the real-world cohort included patients with DLBCL diagnosed between January 1, 2011 and December 31, 2019. Patients were eligible for inclusion if they had at least one FMI comprehensive genomic profiling test before or within 30 days of first-line treatment (*n* = 59). Patients with primary DLBCL of the central nervous system were excluded (*n* = 6). The final real-world DLBCL cohort included 53 patients (Supplementary Methods, online only).

### Biomarker analyses

COO assays were performed by Covance Inc./LabCorp (Princeton, NJ, USA) and determined using the NanoString Lymphoma Subtyping Research-Use-Only assay (NanoString Technologies Inc., Seattle, WA, USA).

BCL2 protein expression was determined by a central laboratory using the Ventana investigational-use only IHC assay (Hematogenix, Ventana Medical Systems, Tucson, AZ, USA; Supplementary Methods, online only). BCL2 positivity was defined as moderate (2+) or strong (3+) staining in ≥50% of tumor cells. BCL2 negativity was defined as no (0) or weak (1+) staining.

### Mutational analysis using next-generation sequencing

RNA was isolated and purified from pre-treatment formalin-fixed, paraffin-embedded tissue sections (FFPE) using the FFPE RNEasy preparation kit (QIAGEN, Carlsbad, CA, USA). The purified RNA was used to create cDNA libraries that were assayed using TruSeq® RNA-Seq (Illumina, San Diego, CA, USA) to measure gene expression in 553 patients with DLBCL in the GOYA study. Further details of RNA-Seq post processing is provided (Supplementary Methods, online only).

**Fig. 1:**
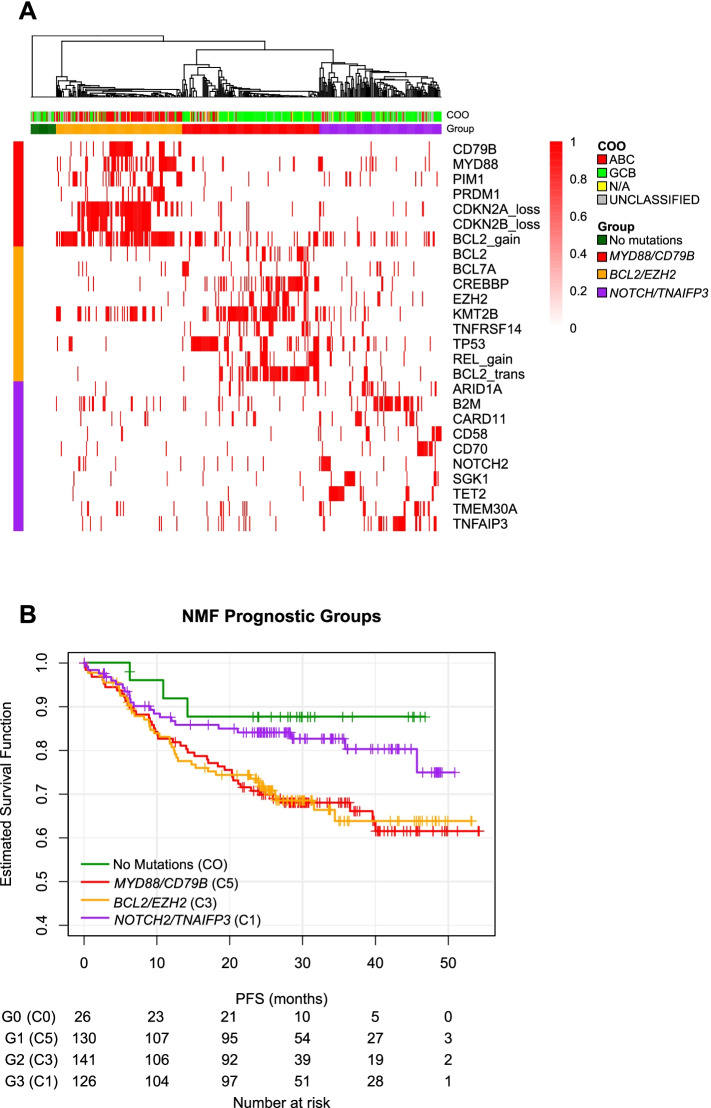
NMF-defined clusters (5% prevalence cut-off) in GOYA and their association with cell-of-origin and Kaplan-Meier curves of individual groups. **A** Clustering by patient into four groups: *CD79B/MY*D88 (red), *BCL2/EZH2* (orange), *NOTCH2/TNAIFP3* (purple), and a group with no mutations (green). **B** Kaplan-Meier curves of PFS for the four NMF prognostic groups. Genomic features represent mutations, copy number alterations (loss/gain), and translocations (trans) as binary values, and are assigned to groups by Fisher’s test (*p* < 0.1 cut-off). COO association is displayed on the top track. No additional stratification according to treatment arm was performed (R-CHOP + G-CHOP). COO, cell-of-origin; NMF, non-negative matrix factorization; PFS, progression-free survival

The F1H panel of 465 genes was used to define single nucleotide variants, copy number amplifications, rearrangements and homozygous deletions. All genomic features variants assessed were those known to be or likely to be pathogenic. Mutations were transformed into a binary representation of presence or absence, regardless of repeated occurrences per subject. A 5% prevalence cut-off was used as the criterion for inclusion for clustering, resulting in the identification of 26 genes with genetic variance in GOYA and 30 genes with genetic variance in CAVALLI (GOYA genes were used for the CAVALLI Random Forest model) (Fig. 1A). Data were available for 423 patients in the GOYA trial and for 86 patients in the CAVALLI trial.

### Statistical analysis

PFS and OS were assessed using Kaplan-Meier survival analysis. Hazard ratios (HRs) with 95% confidence intervals (CI) were calculated using the Cox proportional hazards regression model. Of note, the real-world cohort could not be assessed for PFS using the same criteria as GOYA and CAVALLI due to different frequencies and timings of response assessments across clinics, as well as differing levels of evidence in determining progressive disease. Therefore, only OS was analyzed. Multivariate models were adjusted for treatment arm (GOYA study only), age (continuous), sex, IPI, COO, and BCL2 IHC status. In the real-word multivariate analysis IPI was not included.

Differential gene expression was performed and stratified according to NMF-derived high-/low-risk groups using counts-per-million normalized counts (R-package Limma-Voom, with no covariate adjustment). T-statistic values from Limma were used along with the Molecular Signature Database gene set references for fast gene-set enrichment analysis (R-package). Multiple testing adjustment for RNA-Seq differential expression was done by estimating false discovery rates (FDRs) using the Benjamini-Hochberg procedure (significance <5% FDR).

### Non-negative matrix factorization

NMF clustering was performed according to the method used by Chapuy and colleagues [2] using the NMF R package described by Gaujoux and Seoighe [22]. This analysis was restricted to genes available within the F1H panel and we excluded silent single nucleotide variants and rearrangements. Group sizes (*k*) from 2 through 10 were searched in every case, with the final *k* being chosen by a maximal cophenetic coefficient criterion although recent advances to identify informed group sizes with domain-defined rules and guidelines appear promising (final *K:* GOYA=3, CAVALLI=2) [23, 24]. Genomic features (mutations, copy number alterations, and translocations) were given binary values and their association with each group was performed according to Chapuy et al. A Fisher’s test (*P* < 0.1 cut-off) was used to identify the group with the strongest association to a given gene feature. Equal weight was assigned across alteration types.

### Random Forest modelling

To enable a distinction to be made between patients with a more favorable prognostic outcome and those who responded to venetoclax, a Random Forest classifier was trained on NMF cluster labels and genomic features in GOYA (Supplementary Methods, online only).

## Results

Baseline disease characteristics were similar between patients in the biomarker-evaluable population and the ITT population for both the GOYA and CAVALLI study (Supplementary Table S1).

### NMF-defined clusters

We hypothesized that leveraging the F1H targeted assay would permit the identification of similar molecular subgroups as reported by Chapuy et al. [2] To test this, we applied NMF clustering on *de novo* DLBCL patient samples from the GOYA and CAVALLI studies and the FH-FMI CGDB. In GOYA, three clusters were identified (397 samples): *BCL2/EZH2 Chapuy C3-like* (*n* = 141), *MYD88/CD79B C5-like* (*n* = 130), and *NOTCH2/TNFAIP3 C1-like* (*n* = 126). Samples (*n* = 26) without any of the genetic drivers were assigned to a ‘no mutation’ cluster (Fig. 1A). In CAVALLI, two clusters were identified: *MYD88/CD79B* (*n* = 40), *BCL2/EZH2* (*n* = 40), as well as a group with ‘no mutations’ (*n* = 6); a *NOTCH2/TNAIFP3-like* cluster was not identified (Supplementary Fig. S1). Within the FH-FMI CGDB (53 samples) three clusters were identified: *BCL2/EZH2* (*n* = 25), *BCL2* Amplification/*CDKN2A/B* (*n* = 9)*, CD70/SGK1* (*n* = 16), as well as a group with ‘no mutations’ (*n* = 3).

Generally, the identified subgroups shared similar mutational profiles with clusters identified by Chapuy et al. In GOYA, the *BCL2/EZH2* cluster most closely maps to Chapuy’s C3, *MYD88/CD79B* to C5, *NOTCH2/TNFAIP3* to C1 and ‘no mutations’ to C0. However, there was inconsistency with Chapuy’s defined cluster C2 (*TP53*/Copy-number alteration) and Chapuy C4 (*PI3K/NFKB*) in all cohorts*.* Chapuy’s C2 (*TP53*/Copy-number alteration) could not be recapitulated due to the absence of genome-wide copy number alterations in the targeted F1H panel; *TP53* mutants clustered with *BCL2/EZH2* instead. In contrast, despite sufficient variant coverage in F1H to capture Chapuy’s C4, this cluster was not observed. Clusters identified in CAVALLI were comparable to GOYA except the *NOTCH2/TNAIFP3* group was not present in CAVALLI. Real-world FH-FMI CGDB-derived clusters were similar to those observed in GOYA, however, several key genes were missing in all groups.

Additionally, the distribution of COO subsets with the clusters identified by Chapuy et al correlated well with the same clusters identified by our study. In GOYA, a relatively high proportion of tumors in *MYD88/CD79B* C5-like clusters for all cohorts were ABC-DLBCL type (*n* = 87/130 [67%]). *BCL2/EZH2* C3-like and *NOTCH2/TNAIPF3* C1-like clusters, when present, were predominantly GCB-DLBCL type (*n* = 107/141 [76%] and *n* = 83/126 [66%], respectively).

### Prognostic groups

We investigated whether there were differences in PFS between molecularly defined subgroups. In the GOYA cohort, there was no difference in PFS between the *BCL2/EZH2* and *MYD88/CD79B* clusters, or between the *NOTCH2/TNAIFP3* and ‘no mutation’ clusters (Fig. 1B). Consistent with the prognostic trends reported by Chapuy et al, the *BCL2/EZH2* and *MYD88/CD79B* clusters (equivalent to Chapuy C3 and C5) identified in the GOYA cohort were associated with worse prognosis compared with the ‘no mutations’ and *NOTCH2/TNFAIP3* clusters (equivalent to Chapuy C0 and C1, respectively).

**Fig. 2:**
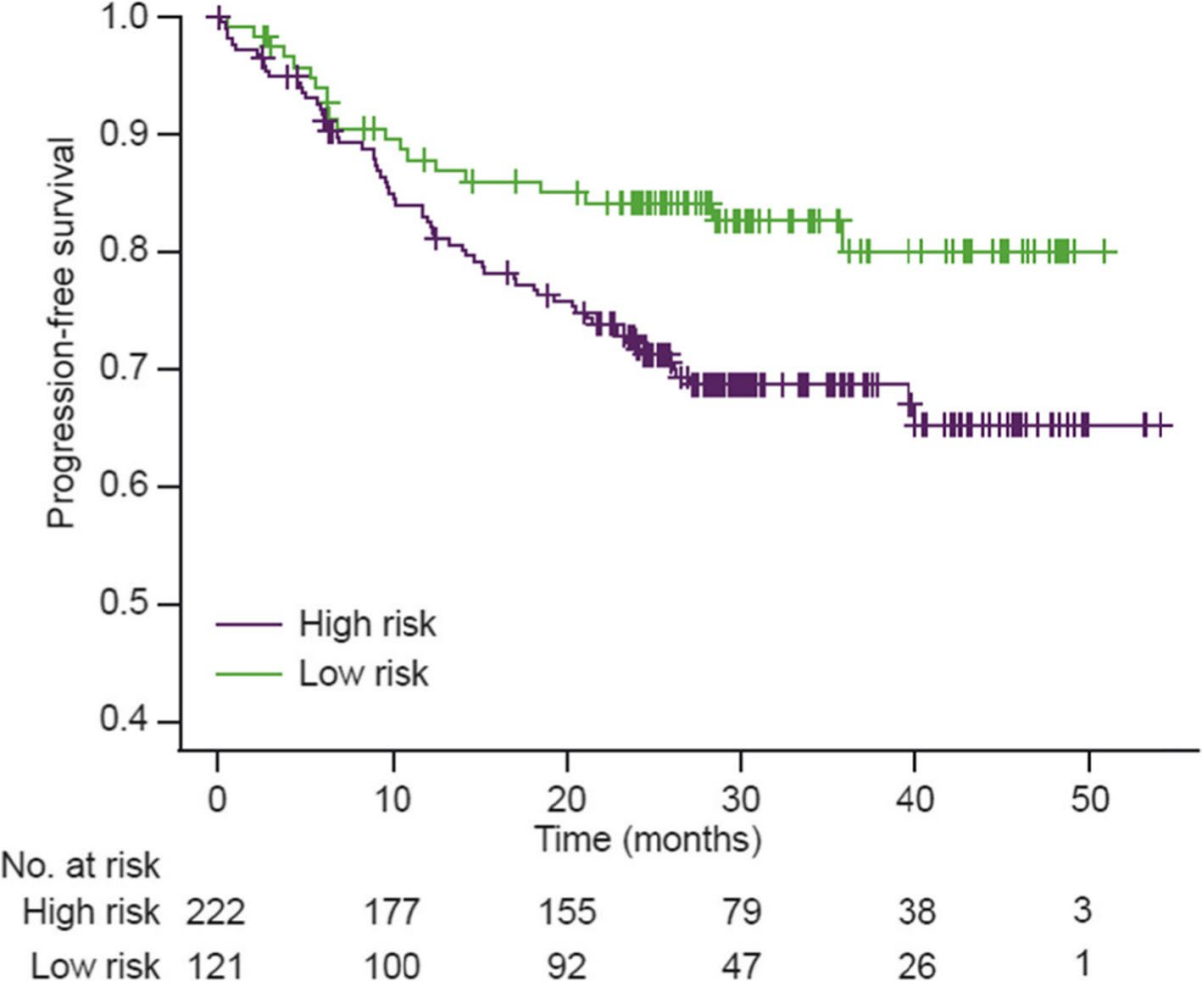
Dichotomized risk model of NMF groups and adjusted multivariate model. PFS of merged groups with similar prognostic profiles into a high-risk group (purple, *CD79B/MYD88* and *BCL2/EZH2*), and low-risk group (green, *NOTCH2/TNAIFP3* and ‘no mutations’). NMF, non-negative matrix factorization; PFS, progression-free survival

Based on these two distinct groupings, the *BCL2/EZH2* and *MYD88/CD79B* clusters were combined into a high-risk subgroup, and the *NOTCH2/TNFAIP3* and ‘no mutations’ clusters were combined into a low-risk subgroup (PFS: HR, 1.62; 95% CI, 0.93-2.83; OS: HR, 1.96 95% CI, 0.97-3.94) (Fig. 2). To test the prognostic value of the high-risk molecularly defined clusters, a multivariate Cox regression analysis was performed, using established clinical predictors, including IPI, as covariates. Although molecular risk remained associated, after adjustment for other covariates, it failed to outperform IPI; instead, molecular risk showed a similar prognostic value to COO. Through further stratification by treatment and COO, we observed prognostic trends between molecular subgroups. In particular, we observed differences in PFS between molecular subgroups in the patients who received R-CHOP (HR, 2.41; 95% CI, 0.99-5.86) and in the patients with GCB DLBCL (HR, 2.04; 95% CI, 1.00-4.19) (Supplementary Fig. S2).

### BCL2 IHC

**Fig. 3:**
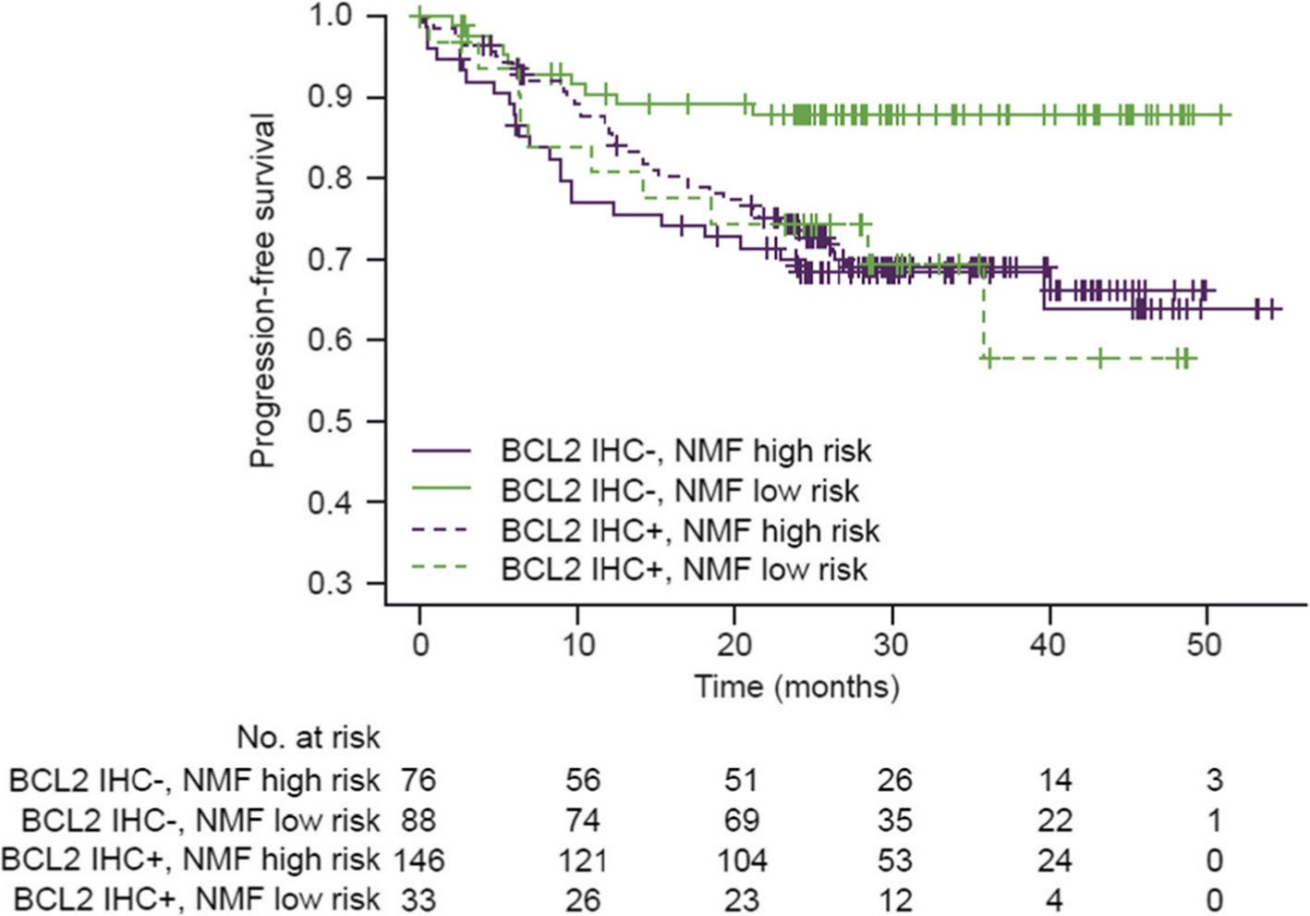
BCL2-related features in NMF risk groups in GOYA. PFS of BCL2 IHC status (IHC status 0,1 as negative, 2,3 as positive) stratified by NMF risk high/low. IHC, immunohistochemistry; NMF, non-negative matrix factorization; PFS, progression-free survival

BCL2 protein expression can be influenced by mutations, copy number amplifications or structural variants, which are components of several of the identified NMF clusters in this study. To understand the contribution of BCL2 in the identified NMF-defined clusters, BCL2 protein expression by IHC was assessed. Patients with BCL2 IHC positive (BCL2*+*) disease were enriched in high-risk groups in GOYA (*MYD88/CD79B* and *BCL2/EZH2*) (odds ratio [OR], 5.08; 95% CI, 3.08-8.54) and CAVALLI (*MYD88/CD79B*) (OR, 1.99; 95% CI, 0.63-6.55), these data were not available in the FH-FMI CGDB. In GOYA, 42% and in CAVALLI, 31% of high-risk patients had BCL2+ disease. BCL2 expression further stratifies NMF-defined low-risk patients, with BCL2+ patients having a poorer prognosis compared with BCL2- patients (PFS: BCL2*+* HR, 2.66; 95% CI, 1.04-6.78; OS: BCL2*+* HR, 1.86; 95% CI, 0.55-6.31; Fig. 3). In contrast, no association between BCL2 expression and outcome was observed in NMF-defined high-risk patients (PFS: BCL2*+* HR, 0.76; 95% CI, 0.45-1.28; OS: BCL2*+* HR, 0.55; 95% CI, 0.29-1.05).

### CAVALLI

**Fig. 4:**
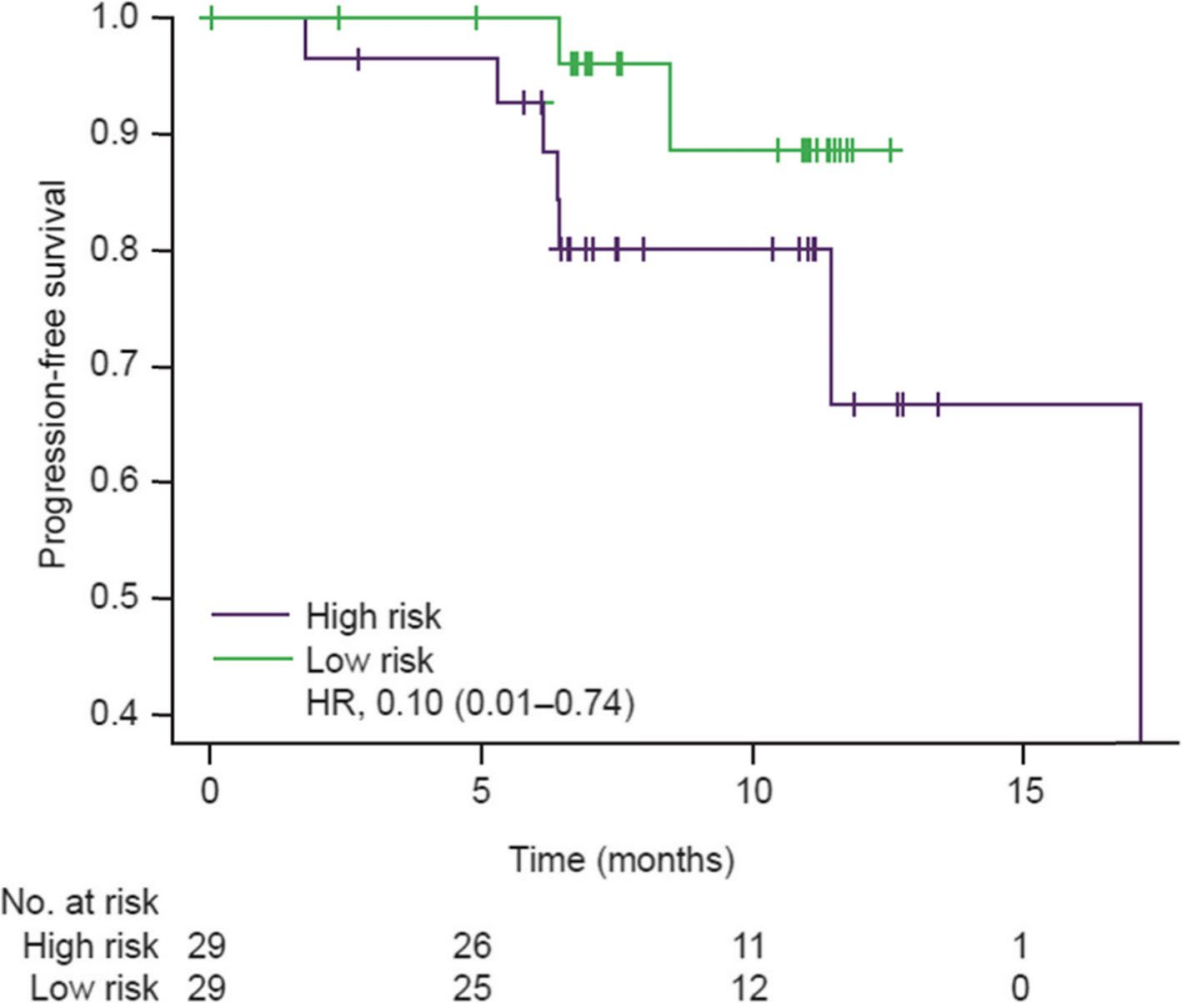
Kaplan-Meier curves of PFS for NMF prognostic groups in CAVALLI categorized as high-risk (*MYD88/CD79B* cluster) and low-risk (*BCL2/EZH2* and ‘no mutations’ clusters). HR, hazard ratio; NMF, non-negative matrix factorization; PFS, progression-free survival

While the genomic features in the *MYD88/CD79B* and *BCL2/EZH2* clusters in CAVALLI bear strong similarity to the clusters in GOYA, PFS and OS outcomes differed from GOYA in the *BCL2/EZH2* cluster (Supplementary Fig. S3). In contrast to GOYA, there was a trend towards improved survival over *MYD88/CD79B* in the *BCL2/EZH2* cluster (PFS: HR, 0.11, 95% CI, 0.01-1.11; OS: HR, 0.12; 95% CI, 0.01-1.58). Moreover, multivariate Cox proportional hazards modelling, using age, sex, IPI, COO, and BCL2 IHC status as covariates, verified that the MYD88/CD79B cluster was associated with poor prognosis compared with the BCL2/EZH2 cluster and ‘no mutations’ (PFS: HR, 0.10; 95% CI, 0.01-0.74; *P* = 0.025) (Fig. 4).

### Random Forest modelling

To control for small sample sizes and potential differences in the NMF data-dependent algorithm, we trained a Random Forest prediction model on GOYA NMF labels (see Methods). Predicting labels on CAVALLI reintroduced the *NOTCH2/TNAIFP3* cluster (Supplementary Fig. S4A). PFS results for the Random Forest-generated groups in CAVALLI were qualitatively similar to those obtained in CAVALLI using the *de novo* NMF model. Of note, the *BCL2/EZH2* cluster followed a similar trend to the *NOTCH2/TNAIFP3* group for PFS (Supplementary Fig. S4B).

## Discussion

In recent years, a number of groups have made strides in capturing the heterogeneous genetic characteristics of DLBCL using complex multi-platform analyses [2, 16, 18], driving the need for simplified methods that enable clinical implementation and inform targeted therapeutic strategies. We applied NMF clustering to targeted exome-sequencing data utilizing the F1H panel on samples obtained from the Phase III GOYA (randomized R-CHOP versus G-CHOP) and Phase Ib/II CAVALLI (venetoclax + R-CHOP) clinical studies. Analysis of data from the GOYA study, in patients with *de novo* DLBCL, demonstrated that the mutation profiles, COO subset distribution, and NMF clusters previously identified using whole exome profiling [2–5, 17] could largely be recapitulated using targeted mutational data from the F1H panel. The NMF clusters were also consistent with subsets identified using the probabilistic classification tool [18]. Importantly, these analyses validate the Chapuy NMF clusters [2] and their prognostic value for standard of care using large, independent, contemporary datasets.

While *BCL2/EZH2* (C3, EZB), *MYD88/CD79b* (C5, MCD), and *NOTCH/TNFAIP3* (C1, BN2) subset identification and effects on prognosis are generally consistent in GOYA compared with the consolidated datasets used in Chapuy et al [2] and Wright et al [18], the panel is limited in terms of copy number amplification measurements, making detection of NMF clusters based on these types of variants (C2, A53) less likely. Consistent with this limitation, *TP53* could not be assigned to the C2-like group due to its absence. Given the effects on prognosis of *TP53* and the potential to target these variants, this is an identified limitation of this simplified platform. Similarly, C4 (ST2) was not observed despite reasonable coverage of genes encoding histone core and linker genes, as well as signaling genes on the F1H panel. A potential reason for the lack of C4 identified in GOYA is that the C4 heterogeneous cluster is defined by at least three mechanistic molecular profiles; histone regulators, signaling pathways and immune evasion. Unlike the other clusters containing key driver gene profiles, this subset is more heterogeneous and therefore may be more difficult to consistently capture. More generic differences could be attributed to variations in the patient populations and treatments with GOYA being more contemporary and homogeneous.

Furthermore, there were differences between GOYA, CAVALLI and FH-FMI CGDB across key genes likely due to reduced sample sizes. While *BCL*2 translocations and *EZH2* variants were present, *BCL2* SNVs were not assigned within the *BCL2/EZH2* cluster in CAVALLI. Additionally, within the FH-FMI CGDB clusters, several key genes were not significantly assigned to clusters of patients. For example, *MYD88, CD79B, NOTCH2* and *TNFAIP3* were missing due to low prevalence and small sample size.

Given the heterogeneous mutational profile of *de novo* DLBCL [1, 16] differences between this analysis and other datasets (i.e. Chapuy) are not altogether unexpected. In contrast to Chapuy et al [2] who reported that genomic clusters were prognostic when adjusted with several clinical variables (e.g. IPI), our study indicated that NMF was not, and was of similar prognostic value to COO. Potentially, the more contemporary GOYA dataset (data cut-off January 31, 2018 vs CAVALLI data cut-off June 28, 2019) and more homogeneous treatment regimen (single trial compared with those for Chapuy, where 85% of patients were uniformly treated with R-CHOP) may account for these differences.

One of the key findings of the NMF clustering analysis using the GOYA and CAVALLI datasets is that while we were able to identify and confirm the prognostic potential of the high-risk NMF subsets (*BCL2/EZH2* and *MYD88/CD79B*) to standard of care, validation of these NMF-defined subsets using the CAVALLI dataset showed distinct clinical response profiles; *MYD88/CD79B* exhibits high-risk/poor clinical response to the venetoclax R-CHOP regimen while in contrast, the *BCL2/EZH2* subset appears to benefit and thus may be predictive for targeted therapies such as venetoclax. This difference in PFS outcome between GOYA and CAVALLI (Supplementary Fig. S5) is potentially explained by the mechanism of action of venetoclax, a highly selective BCL2 inhibitor [19], and its potential to improve treatment outcomes for patients with DLBCL with elevated levels of *BCL2* [19, 20, 25, 26]. However, there are multiple other reasons this could occur and further confirmation is necessary. The addition of venetoclax to standard of care immunochemotherapy resulted in categorization of the *BCL2/EZH2* cluster as low-risk; while the *MYD88/CD79B* cluster is maintained as high-risk across therapeutic settings. However, as the *MYD88/CD79B* cluster also includes *BCL2* amplifications, we cannot explain why venetoclax might specifically benefit patients with either *BCL2* mutations or translocations over amplifications. These data also highlight the limitations of BCL2 IHC alone as insufficient to define prognosis. Interestingly, elevated BCL2 levels by IHC may further stratify low-risk NMF clusters into high- and low-risk categories. In these clusters, different mechanistic drivers of BCL2 expression and activation are again present (mutations, amplifications and translocations) and likely contribute to prognostic or predictive potential warranting further investigation.

The real-world data cohort had the greatest differences compared with GOYA, where *MYD88* and *CD79B* mutations had low prevalence (9/53 patients and 6/53 patients, respectively), *NOTCH2* mutations were detected in only two patients and *TNFAIP3* were detected in 8/53 patients. We attempted to corroborate our findings in a real-world setting, but differences in the prevalence of key variants and small sample size limited interpretation.

## Conclusions

In summary, the aim of our study, was to identify a simplified platform for the identification of molecularly-defined subsets of DLBCL to inform treatment in clinical practice. Using the F1H assay, we were able to recapitulate previously defined prognostic subsets in contemporary datasets and demonstrate the potential to identify molecular subsets sensitive to targeted rational therapies, such as venetoclax, that require further validation.

## Supplementary Information

**Additional file 1: Supplementary Methods and Results. Supplementary Table S1.** Baseline disease characteristics of patients in the BEP and ITT study populations in GOYA and CAVALLI. **Supplementary Table S2.** Confusion matrix for Random Forest training model. **Supplementary Table S3.** A. CAVALLI ethics committees (EC) and/or institutional review boards (IRB) of participating centers. B. GOYA ethics committees (EC) and/or institutional review boards (IRB) of participating centers. **Supplementary Figure S1.** NMF-defined clusters in CAVALLI and their association with cell-of-origin. **Supplementary Figure S2.** Kaplan-Meier curves of PFS for NMF high- and low-risk prognostic groups in GOYA according to (A) treatment and (B) cell-of-origin (activated B-cell-like vs germinal center B-cell-like). **Supplementary Figure S3.** Kaplan-Meier curve of PFS for the *BCL2/EZH2* versus *MYD88/CD798* clusters in CAVALLI (*de novo* NMF clustering). **Supplementary Figure S4.** Training of a Random Forest model on NMF cluster labels in GOYA onto CAVALLI. (A) Clustering of gene features of predicted GOYA NMF groups onto CAVALLI. (B) Kaplan-Meier curves of PFS for predicted GOYA NMF groups in CAVALLI. **Supplementary Figure S5.** Kaplan-Meier curves of PFS for GOYA and CAVALLI. **Supplementary Figure S6.** Differential expression and pathway analysis of NMF highrisk vs low-risk patients in GOYA. (A) Volcano plot of all genes for patients with NMF risk categorization and RNA-Seq data. Limma was used to determine differential expression with no covariate adjustment. Labelled points outside of the red dotted lines indicate genes with a false discovery rate <0.05 (Benjamini-Hochberg) and log-fold change >1. (B) Pathways with false discovery rate <0.05 using fast gene set enrichment analysis. tStat values from Limma were used along with the mSigDB Hallmark signature list. (C) Top 30 ranked normalized enrichment scores for Staudt Signature database. All signatures have adjusted p-values with a false discovery rate <0.05. (D) Additional signature sets from MSigDB.

## Data Availability

Qualified researchers may request access to individual patient level data through the clinical study data request platform (https://vivli.org/). Further details on Roche's criteria for eligible studies are available here (https://vivli.org/members/ourmembers/). For further details on Roche's Global Policy on the Sharing of Clinical Information and how to request access to related clinical study documents, see here: https://www.roche.com/research_and_development/who_we_are_how_we_work/clinical_trials/our_commitment_to_data_sharing.htm The real-world data that support the findings of this study have been originated by Flatiron Health, Inc. and Foundation Medicine Inc. These de-identified data may be made available upon request, and are subject to a license agreement with Flatiron Health and Foundation Medicine; interested researchers should contact <cgdb-fmi@flatiron.com> to determine licensing terms.

## Abbreviations:

DLBCL: Diffuse large B-cell lymphoma
NMF: Non-negative matrix factorization
CHOP: Cyclophosphamide, doxorubicin, vincristine, and prednisone
R: Rituximab
G: Obinutuzumab
HR: Hazard Ratio
CI: Confidence interval
COO: Cell-of-origin
ABC: Activated B-cell-like
GCB: Germinal center B-cell-like
OS: Overall survival
PFS: Progression-free survival
ITT: Intent-to-treat
FMI: Foundation Medicine Inc.
IHC: Immunohistochemistry
IPI: International Prognostic Index
