# Supplementary material for: Prognostic mutational subtyping in de novo diffuse large B-cell lymphoma
Source: BMC Cancer. 2022 Mar 3;22:231. doi: 10.1186/s12885-022-09237-5 (PMC8892802; doi:10.1186/s12885-022-09237-5)
Supplement: Supplementary file 1 — Additional file 1: Supplementary Methods and Results. Supplementary Table S1. Baseline disease characteristics of patients in the BEP and ITT study populations in GOYA and CAVALLI. Supplementary Table S2. Confusion matrix for Random Forest training model. Supplementary Table S3. A. CAVALLI ethics committees (EC) and/or institutional review boards (IRB) of participating centers. B. GOYA ethics committees (EC) and/or institutional review boards (IRB) of participating centers. Supplementary Figure S1. NMF-defined clusters in CAVALLI and their association with cell-of-origin. Supplementary Figure S2. Kaplan-Meier curves of PFS for NMF high- and low-risk prognostic groups in GOYA according to (A) treatment and (B) cell-of-origin (activated B-cell-like vs germinal center B-cell-like). Supplementary Figure S3. Kaplan-Meier curve of PFS for the BCL2/EZH2 versus MYD88/CD798 clusters in CAVALLI (de novo NMF clustering). Supplementary Figure S4. Training of a Random Forest model on NMF cluster labels in GOYA onto CAVALLI. (A) Clustering of gene features of predicted GOYA NMF groups onto CAVALLI. (B) Kaplan-Meier curves of PFS for predicted GOYA NMF groups in CAVALLI. Supplementary Figure S5. Kaplan-Meier curves of PFS for GOYA and CAVALLI. Supplementary Figure S6. Differential expression and pathway analysis of NMF highrisk vs low-risk patients in GOYA. (A) Volcano plot of all genes for patients with NMF risk categorization and RNA-Seq data. Limma was used to determine differential expression with no covariate adjustment. Labelled points outside of the red dotted lines indicate genes with a false discovery rate <0.05 (Benjamini-Hochberg) and log-fold change >1. (B) Pathways with false discovery rate <0.05 using fast gene set enrichment analysis. tStat values from Limma were used along with the mSigDB Hallmark signature list. (C) Top 30 ranked normalized enrichment scores for Staudt Signature database. All signatures have adjusted p-values with a false discovery rate <0.05. (D) [file 12885_2022_9237_MOESM1_ESM.pdf]

Supplementary Methods and Results

GOYA inclusion criteria

Included patients had previously untreated, histologically documented, CD20-positive diffuse large B-cell lymphoma (DLBCL); Eastern Cooperative Oncology Group (ECOG) performance status of 0-2; and International Prognostic Index (IPI) score ≥2. Patients with an IPI score of 1 and aged ≤60 years, with or without bulky disease, and those with an IPI score of 0 and bulky disease (i.e. one lesion ≥7.5 cm) were also included.

CAVALLI inclusion criteria

Included patients had previously untreated, histologically documented B-cell non-Hodgkin lymphoma; at least one bi-dimensionally measurable lesion on computed tomography scan defined as >1.5 cm in its longest dimension; ECOG performance status of 0-2; and IPI score 2-5.

Real-world data analysis

Flatiron Health-Foundation Medicine Inc clinico-genomic database (FH-FMI CGDB)

De-identified patient-level clinical data from the electronic health record included structured data (e.g. laboratory values, prescribed drugs) and unstructured data (e.g. detailed biomarkers) collected via technology-enabled chart abstraction of physician’s notes and other unstructured documents. De-identified patient-level genomic data included specimen features (e.g. tumor mutation burden, pathologic purity), alteration-level details (e.g. genomic position, reference and alternate alleles, mutant allele count, minor allele frequency), and therapeutic recommendations that were reported to the clinician at the time of testing. Genomic alterations were identified via comprehensive genomic profiling of >300 cancer-related genes on FMI's next-generation sequencing-based FoundationOne® panel. To date, more than 400,000 samples have been sequenced from patients across the US.

Institutional Review Board approval of the study protocol was obtained prior to study conduct. Informed consent was waived as this was a non-interventional study and the anonymized data in the FH-FMI CGDB are protected against breach of confidentiality.

Data analysis

Only genes that were in the gene clusters identified in GOYA were selected for the analysis. Mutation test results were preprocessed using the same data preparation methods as already described (i.e. mutations were aggregated per gene level with a binary representation of presence or absence, regardless of types and number of mutations on the gene).

The number of clusters and the associations between the genes and the clusters were determined by non-negative matrix factorization (NMF) using the same procedure described in the methods section. Data to derive disease progression based on change in tumor dimensions were not available in the real-world cohort, and other surrogate definitions of disease progression such as change in line of therapy, were also not yet validated. Therefore, survival analysis for each genetic group was only applied with overall survival as the endpoint. Survival time was measured from the initiation of first-line treatment until death. All patients without a death event were censored at the last visit date or last treatment administration date (whichever came last) on or before the end of the study period (December 2019). Kaplan-Meier curves were used to evaluate overall survival from treatment initiation by treatment regimen. Cox proportional hazard models were used to assess the hazard ratio of genetic subgroups with adjustment of age, sex, cell-of-origin (COO) and BCL2 immunohistochemistry (IHC). Due to the high percentage of missing variables, such as ECOG, which were needed for IPI score calculation, we were unable to include IPI score in the multivariate model.

Biomarker analyses

COO classification subgroups were determined for patients with available tissue and centrally confirmed CD20+ DLBCL using the research-use-only version of the NanoString Lymphoma Subtyping Test (LST; NanoString Technologies, Inc., Seattle, USA). Treatment effect was assessed according to the Linear Predictor Score (LPS) from the NanoString LST; this continuous variable provides information on the likelihood of a patient with DLBCL having an activated B-cell-like tumor based on their gene expression profile, and is a standard output of the LST assay.[1] The performance of obinutuzumab (G)-cyclophosphamide, doxorubicin, vincristine, and prednisone (CHOP) vs rituximab (R)-CHOP was evaluated according to LPS quartiles. BCL2 expression (IHC) and *BCL2* and *MYC* translocations (fluorescence *in situ* hybridization [FISH]) were assayed in pretreatment tumor samples from biomarker-evaluable patients.

Vysis LSI Dual Color Break Apart FISH probes (Abbott Laboratories, Chicago, IL, USA) were used to identify *BCL2* and *MYC* translocations, with FISH+ defined as ≥50% of tumor cells harboring break apart signal. Although there is no standard cut-off defined in the literature, a value of 5% is commonly used. The higher FISH cut-off used here was based on the bimodal distribution of patients in GOYA, which showed clear groupings of patients above 50% and below 12%, with a small number of indeterminate patients between these values. Orthogonal translocation data from sequencing analyses showed these indeterminate patients to in fact be non-translocated, which led to selection of a ≥50% cut-off for this population. The 5% cut-off was used for sensitivity analysis

The H-score was calculated for staining of tumor cells as previously reported by Pfeifer et al,[2] using the following formula:

H-Score = (% at 0) × 0 + (% at 1+) × 1+(% at 2+) × 2+(% at 3+) ×3.

RNA-Seq post processing

Quality control of high-throughput transcriptome sequencing was performed. Adapters were trimmed using ea-utils function fastq-mcf (parameters: --max-ns 4 --qual-mean 25 -H -p 5 -q 7 -l 25 -l 25). Trimmed reads were aligned to human genome reference GRCh38 using GSNAP version 2013-10-10 (parameters: -M 2 -n 10 -B 2 -i 1 -N 1 -w 200000 -E 1 --pairmax-rna=200000 --clip-overlap). Exonic gene counts were determined for each sample by leveraging the in-house Genentech RNA-Seq pipeline scripts. Genes and samples were filtered using an internal RNA-Seq processing package (RNAseqTools; parameters: default). To account for sequencing variability, counts were normalized to counts per million. From 553 samples, a total of 538 passed quality control and were used in downstream analyses.

Random Forest modelling

Random Forest modelling was performed via the caret R package, which uses the R package Random Forest. To minimize data set selection biases, one hundred models in GOYA were trained using a random 70% training and 30% holdout/validation scheme. Each individual model was trained using a repeated cross-validation scheme with 5 folds and 5 repeats, and the best mtry hyper-parameter was selected from the range 1 to 15. Overall model performance was assessed with a confusion matrix (**Supplementary Table S2**), and an mtry value of 3 was selected due to greatest frequency of appearance and its strong recapitulation of the original distribution of GOYA NMF groups vis-à-vis the 3-year progression-free survival landmark.

RNA expression

Differential gene expression analysis (Limma-Voom), comparing high-/low-risk molecular subgroups in GOYA, showed 12 genes as significantly upregulated and 30 genes as downregulated in the high-risk group (**Supplementary Fig. S6A**). *BCL2* was the most differentially expressed gene, significantly upregulated in patients in the high-risk groups (*MYD88/CD79* and *BCL2/EZH2* clusters). Among the most downregulated genes in the high-risk group, several encode different isoforms of collagen proteins (COL1A1, COL3A1, COL5A1, COL11A1) and proteins that remodel the extracellular matrix (FAP, MMP1, MMP2,

MXRA5), which are characteristic of the stromal-1 signature of DLBCL.[3] The stromal-1 signature is enriched with genes expressed in normal mesenchymal tissues and in CD14+ blood monocytes.[3] Indeed, fast gene-set enrichment analysis (fGSEA) using lymphoma-specific pathways[4] showed that several stromal-1-, mesenchymal-, monocyte-, and COO-related signatures were significantly enriched in the genes downregulated in high-risk compared with low-risk patients (**Supplementary Fig. S6B**). Similar gene sets were found to be enriched using the Molecular Signature Database Hallmark, C2, and C7 datasets in the fGSEA analysis (**Supplementary Fig. S6C and S6D**).

Supplementary Table S1. Baseline disease characteristics of patients in the BEP and ITT study populations in GOYA and CAVALLI

	GOYA		CAVALLI	
	ITT (n = 1418)	BEP (n = 423)	ITT (n = 267)	BEP (n = 86)
**Median age (range), years**	62 (18–86)	64 (18–86)	64 (18–85)	64 (27–77)
**Male, n (%)**	752 (53.0)	213 (50.4)	146 (54.7)	49 (57.0)
**ECOG PS, n (%)**	n = 1417	n = 422	n = 266	n = 86
0–1	1231 (86.9)	372 (88.2)	229 (86.1)	77 (89.5)
2–3	186 (13.1)	50 (11.9)	–	–
≥2	–	–	37 (13.9)	9 (10.5)
**IPI, n (%)**	n = 1418	n = 423	n = 215	n = 62
Low	283 (20.0)	89 (21.0)	3 (1.4)	1 (1.6)
Low-int	502 (35.4)	147 (34.8)	87 (40.5)	26 (41.9)
High-int	413 (29.1)	116 (27.4)	72 (33.5)	18 (29.0)
High	220 (15.5)	71 (16.8)	53 (24.7)	17 (27.4)
**Elevated LDH, n (%)**	n = 1413	n = 422	n = 265	n = 86
	816 (57.8)	240 (56.9)	149 (56.2)	48 (55.8)
**Treatment, n (%)**	n = 1407	n = 421	n = 267	n = 86
G-CHOP	704 (50.0)	211 (50.1)	32 (12.0)	14 (16.3)
R-CHOP	703 (50.0)	210 (49.9)	232 (86.9)	72 (83.7)
Not treated	–	–	3 (1.1)	0 (0)
**COO, n (%)**	n = 1418	n = 423	n = 212	n = 77
ABC	243 (17.1)	123 (29.1)	56 (26.4)	23 (29.8)
GCB	540 (38.1)	225 (53.2)	124 (58.5)	42 (54.6)

Unclassified	150 (10.6)	62 (14.7)	32 (15.1)	22 (15.6)

ABC, activated B-cell-like; BEP, biomarker-evaluable population; COO, cell-of-origin; ECOG PS, Eastern Cooperative Oncology Group performance status; G-CHOP, obinutuzumab plus cyclophosphamide, doxorubicin, vincristine and prednisone; GCB, germinal center B-cell-like; int, intermediate; IPI, International Prognostic Index; ITT, intent-to-treat; LDH, lactate dehydrogenase; R-CHOP, rituximab plus cyclophosphamide, doxorubicin, vincristine and prednisone.

Supplementary Table S2. Confusion matrix for Random Forest training model

	Predicted, n				
	No mutations (C0)	*MYD88/CD79B* (C5)	*BCL2/EZH2* (C3)	*NOTCH2/TNAIFP3* (C1)	Class error
**Observed, n**					
No mutations (C0)	26	0	0	0	0
MYD88/CD79B (C5)	0	118	8	4	0.0923
BCL2/EZH2 (C3)	3	2	133	3	0.0567
NOTCH2/TNAIFP3 (C1)	1	16	14	95	0.2460

Supplementary Table S3A. CAVALLI ethics committees (EC) and/or institutional review boards (IRB) of participating centers**.**

CAVALLI Study: Site Name	Site Country	IRB/EC Name
Fakultni nemocnice Hradec Kralove	Czech Republic	Eticka komise Fakultni
Hospital Universitario La Paz	Spain	CEIC Hospital Universitario
CHU Montpellier - Hopital Saint Eloi	France	Comité de Protection des Personnes
University of Rochester	United States	WIRB
VU Medisch Centrum	Netherlands	VU Medisch Centrum
The West Clinic	United States	WIRB
Cross Cancer Institute	Canada	Alberta Cancer Research Ethics Board
Azienda Ospedaliero Universitaria San Martino	Italy	Comitato Etico Regionale della
Jonsson Comprehensive Cancer Center	United States	UCLA Medical Center IRB
Semmelweis Egyetem	Hungary	Orszagos Gyogyszereszteti es Élelmezés-egészségügyi
TRIO - Central Coast Medical Oncology Corporation	United States	WIRB
BC Cancer Agency Center for the Southern Interior	Canada	UBC BCCA REB
CHU de Nancy - Hôpital de Brabois Adultes	France	Comité de Protection des Personnes
Tennessee Oncology	United States	WIRB
Concord Repatriation General Hospital	Australia	Sydney Local Health Network
ICO l´Hospitalet – Hospital Duran i Reynals	Spain	CEIC Hospital Universitario
Hackensack University Medical Center PARTNER	United States	WIRB
LKH - Universitätsklinikum der PMU Salzburg	Austria	Ethikkommission der PMU
Hôpital Henri Mondor	France	Comité de Protection des Personnes
Fakultni nemocnice Ostrava	Czech Republic	Eticka komise Fakultni
Debreceni Egyetem	Hungary	Orszagos Gyogyszereszteti es Élelmezés-egészségügyi
AKH - Medizinische Universität Wien	Austria	Ethikkommission der Medizinischen Universität Wien
SMBD Jewish General Hospital d/b/a Jewish General Hospital	Canada	Jewish General Hospital
TRIO - St. Jude Heritage Healthcare	United States	WIRB
CHU Rennes - Hopital Pontchaillou	France	Comité de Protection des Personnes

CHU de Quebec - Hôpital de l' Enfant Jésus	Canada	CHA-Hopital Enfant Jesus-Comite d’éthique de la
Clinique Victor Hugo - Centre Jean Bernard	France	Comité de Protection des Personnes
CHU Nantes - Hôtel Dieu	France	Comité de Protection des Personnes
Hospital Universitario Ramon y Cajal	Spain	CEIC Hospital Universitario Ramon y Cajal
Erasmus Medisch Centrum	Netherlands	VU Medisch Centrum
Centre Hospitalier Départemental Les Oudairies	France	Comité de Protection des Personnes
Hospital Universitari Vall d'Hebron	Spain	CEIC Hospital Universitario
Princess Alexandra Hospital	Australia	Metro South Health Service
Hospital Universitario de Salamanca	Spain	CEIC Hospital Universitario
Peter MacCallum Cancer Centre-East Melbourne	Australia	Peter MacCallum Cancer Centre Research Ethics
Royal Melbourne Hospital	Australia	Melbourne Health Human Research Ethics Committee
Fakultni nemocnice Brno	Czech Republic	Eticka komise Fakultni fakultní nemocnice
UMC Utrecht	Netherlands	VU Medisch Centrum
Hopital Claude Huriez - CHRU Lille	France	Comité de Protection des Personnes
San Juan Oncology Associates	United States	Copernicus Group IRB
Azienda Ospedaliera Vincenzo Cervello	Italy	Comitato Etico Regionale della
Azienda Ospedaliero Universitaria Pisana	Italy	Comitato Etico Area Vasta Nord
Istituto Nazionale Tumori Fondazione G. Pascale	Italy	Comitato Etico Regionale della
Orszagos Onkologiai Intezet	Hungary	Orszagos Gyogyszereszteti es Élelmezés-egészségügyi
Hospital del Mar	Spain	CEIC Hospital Universitario
Centre Hospitalier Lyon Sud	France	Comité de Protection des Personnes
BC Cancer Agency Vancouver Centre	Canada	UBC BCCA REB
Hôpital Saint-Louis	France	Comité de Protection des Personnes
Centre Henri Becquerel	France	Comité de Protection des Personnes
Vseobecna fakultni nemocnice v Praze	Czech Republic	Eticka komise Vseobecne
Azienda Ospedaliera Città della Salute e della Scienza di	Italy	Azienda Ospedaliera Città della Salute e della Scienza di
Memorial Sloan Kettering Cancer Center	United States	Memorial Sloan-Kettering

Supplementary Table S3B. GOYA ethics committees (EC) and/or institutional review boards (IRB) of participating centers

Site #	Investigator	EC/IRB Name and Address	IRB/EC Approval
208081	Viola, Luciana	Comitê de Ética em Pesquisa da PUCRS, Avenida Ipiranga, 6690 – 3º andar – Sala 314, 90610-000, Porto Alegre, RS, BRAZIL	06-Dec-2013
208254	Willenbacher, Ella	Ethikkommission der Medizinischen Fakultaet der Universitaet Wien und des AKH der Stadt Wien, Borschkegasse 8b/E 06, 1090, Wien, AUSTRIA	15-Feb-2013
208711	Jorgensen, Judith	De Videnskabsetiske Komitéer for Region Hovedstade, Kongens Vænge 2, 3400, Hillerød, DENMARK	30-Apr-2013
208815	Brown, Peter	De Videnskabsetiske Komitéer for Region Hovedstade, Kongens Vænge 2, 3400, Hillerød, DENMARK	30-Apr-2013
208827	Bjørn Poulsen, Christian	De Videnskabsetiske Komitéer for Region Hovedstade, Kongens Vænge 2, 3400, Hillerød, DENMARK	30-Apr-2013
208970	JAEGER, ULRICH G.	Ethikkommission der Medizinischen Fakultaet der Universitaet Wien und des AKH der Stadt Wien, Borschkegasse 8b/E 06, 1090, Wien, AUSTRIA	15-Feb-2013
208979	Suh, Jason	Western Institutional Review Board WIRB Panel 7, 1019 39th Ave S.E., Puyallup, WA, 98374, UNITED STATES	19-Jul-2011
208981	Chaves, Jorge Mario	Western Institutional Review Board WIRB Panel 7, 1019 39th Ave S.E., Puyallup, WA, 98374, UNITED STATES	10-May-2011
208984	Lerner, Rachel	Park Nicollett Institute IRB, 3800 PARK NICOLLET BLVD, ST. LOUIS PARK, MN, 55416, UNITED STATES	17-Aug-2011
208988	Liu, Herman Sung-Yu	Ethics Committee, Hong Kong East Cluster, 3 Lok Man Road, Chai Wan, HONG KONG	09-Jan-2013
208989	Kwong, Yok-Lam	HKU/HA HKW IRB, Room 901, Adminstration Block, 102 Pok Fu Lam Road, Queen Mary Hospital, Hong Kong, HONG KONG	09-Nov-2012
209499	CHUNCHARUNEE, SUPORN	Ethical Clearance Committee on Human Rights, 270 RamaVI Road. Faculty of Medicine, Ramathibodi Hospital, Phayathai Rajathevi Bangkok 10400, 10400, Bangkok, THAILAND	13-Jul-2011
209500	SIRITANARATKUL, NOPPADOL	Ethics Committee, Faculty of Medicine, Siriraj Hospital, Mahidol University, 10700, Bangkok, THAILAND	30-May-2011
209517	Bunworasate, Udomsak	Institutional Review Board, Faculty of Medicine, 3rd floor, Ananda Mahidol Building,, 10330, Patumwan, Bangkok, THAILAND	06-Jul-2011
209518	CHANSUNG, KANCHANA	The Khon Kaen University Ethics Committee in human research, 123 Mitraphap Road, Dean Office 6Th Floor, Faculty Of Medicine, Khon Kaen University, 40002, Khon Kaen, THAILAND	13-Jul-2011
209519	ARPORNWIRAT, WICHIT	Ethics Committee, National Cancer Institute, 268/1 Rama VI Road,, Rajathevee,, 10400, Bangkok, THAILAND	23-May-2011

Site #	Investigator	EC/IRB Name and Address	IRB/EC Approval
209519	ARPORNWIRAT, WICHIT	IHRP Ethics Committee; Dept. of Medical Science, Building 8, Floor 7, Room 702-703, Tiwanon Rd., Amphur Muang, Ministry Public Health, 11000, Nonthaburi, THAILAND	23-May-2011
209520	THUNGTHONG, PRAVINWAN	IHRP Ethics Committee; Dept. of Medical Science, Building 8, Floor 7, Room 702-703, Tiwanon Rd., Amphur Muang, Ministry Public Health, 11000, Nonthaburi, THAILAND	23-May-2011
209520	THUNGTHONG, PRAVINWAN	The Ethics Committee, Rajavithi Hospital, 2 Phyathai Rd. Rajthevee, 10400, Bangkok, THAILAND	23-May-2011
209625	Dyer, Martin	Leicester Royal Infirmary; Research & Development;, Gwendolen House, Gwendolen Road, Leicester, Leicester, LE5 4QF, UNITED KINGDOM	17-Oct-2011
209632	Theocharides, Alexandre	Kantonale Ethikkommission Zürich (KEK), Kantonale Ethikkommission, Stampfenbachstrasse 121, 8090, Zürich, SWITZERLAND	29-Jun-2011
209638	Mamot, Christoph	Ethikkommission Nordwest- und Zentralschweiz (EKNZ), Hebelstrasse 53, 4056, Basel, SWITZERLAND	28-Jun-2011
209639	Mey, Ulrich	Kantonale Ethikkommission Zürich (KEK), Kantonale Ethikkommission, Stampfenbachstrasse 121, 8090, Zürich, SWITZERLAND	29-Jun-2011
209640	ZUCCA, EMANUELE	Comitato Etico Cantonale, Via Orico 5, 6501, Bellinzona, SWITZERLAND	21-Apr-2011
209663	Culligan, Dominic	Aberdeen R&D Office; R&D Office, Foresterhill House Annexe, Ashgrove West, Foresterhill, Aberdeen, AB25 2ZB, UNITED KINGDOM	04-Oct-2011
209667	Basu, Supratik	Wolverhampton Local Research Ethics Committee, Wolverhampton City PCT, Coniston House, Chapel Ash, Wolverhampton, WV3 0XE, UNITED KINGDOM	17-Oct-2011
209728	GREIL, RICHARD	Ethikkommission der Medizinischen Fakultaet der Universitaet Wien und des AKH der Stadt Wien, Borschkegasse 8b/E 06, 1090, Wien, AUSTRIA	15-Feb-2013
209920	RUFF, PAUL	University of the Witwatersrand Research EC, 8 Blackwood Avenue, Parktown 2193, Postnet Suite 189, Private Bag x2600, Houghton, 2041, Johannesburg, SOUTH AFRICA	26-Apr-2013
209948	Follows, George	Addenbrookes Hospital; R&D Department, Block S4, Box 277, Hills Road, Cambridge, CB2 0QQ, UNITED KINGDOM	02-Aug-2011
209949	Paneesha, Shankara	HeartofEngland NHS Foundation Trust; Research and Development Directorate, MIDRU, Birmingham Heartlands Hospital, Brodesley Green East, Birmingham, B9 5SS, UNITED KINGDOM	13-Oct-2011
209977	Quiel, Dimas	Comité Nacional de Bioética Inst.Conmem. Gorgas, Avenida Justo Arosamena y Calle 35, Inst. Conmemorativo Gorgas de Estudio de la Salud, 0816-02593, Panama, PANAMA	21-Jan-2014

Site #	Investigator	EC/IRB Name and Address	IRB/EC Approval
210038	Gironella Mesa, Mercedes	CEIC Hospital Vall D'Hebron, PASSEIG DE LA VALL D'HEBRON S/N 119-129, 08035, BARCELONA, BARCELONA, SPAIN	08-Apr-2011
210039	De La Cruz Merino, Luis	CEIC Hospital Vall D'Hebron, PASSEIG DE LA VALL D'HEBRON S/N 119-129, 08035, BARCELONA, BARCELONA, SPAIN	08-Apr-2011
210039	De La Cruz Merino, Luis	CEIC Virgen Macarena, Avenue Dr. Fedriani, 3 - Investigation Department 2nd Floor, 41009, Sevilla, SEVILLA, SPAIN	08-Apr-2011
210040	Alvarez, Ruth	CEIC Hospital Vall D'Hebron, PASSEIG DE LA VALL D'HEBRON S/N 119-129, 08035, BARCELONA, BARCELONA, SPAIN	09-Nov-2012
210040	Alvarez, Ruth	Comité Ético de Investigación Clínica, Hospital Virgen de la Salud, Avda Barber 30, 45004, Toledo, TOLEDO, SPAIN	09-Nov-2012
210041	Lopez Jimenez, Javier	CEIC Hospital Vall D'Hebron, PASSEIG DE LA VALL D'HEBRON S/N 119-129, 08035, BARCELONA, BARCELONA, SPAIN	08-Apr-2011
210041	Lopez Jimenez, Javier	Comite Ético de Investigación Clínica Agencia de Ensayos-HOSPITAL RAMÓN Y CAJAL, CTRA. DE COLMENAR VIEJO, KM 9,1, 28034, MADRID, MADRID, SPAIN	08-Apr-2011
210045	Gonzalez Barca, Eva	CEIC Hospital de Bellvitge, C/ FEIXA LLARGA, S/N 08907, HOSPITALET DE LLOBREGAT, 08907, BARCELONA, BARCELONA, SPAIN	08-Apr-2011
210045	Gonzalez Barca, Eva	CEIC Hospital Vall D'Hebron, PASSEIG DE LA VALL D'HEBRON S/N 119-129, 08035, BARCELONA, BARCELONA, SPAIN	08-Apr-2011
210046	Salar Silvestre, Antonio	CEIC Hospital Vall D'Hebron, PASSEIG DE LA VALL D'HEBRON S/N 119-129, 08035, BARCELONA, BARCELONA, SPAIN	08-Apr-2011
210046	Salar Silvestre, Antonio	CEIC Parc de Salut Mar; IMIM- Hospital del Mar, C/ Dr. Aiguader, 88, Planta 1, 08003, Barcelona, BARCELONA, SPAIN	08-Apr-2011
210047	Goñi, Mª Angeles	CEIC de Navarra; Departamento de Salud. Pabellón de Docencia., C/ Irunlarrea, 3, 31008, Pamplona, NAVARRA, SPAIN	08-Apr-2011
210047	Goñi, Mª Angeles	CEIC Hospital Vall D'Hebron, PASSEIG DE LA VALL D'HEBRON S/N 119-129, 08035, BARCELONA, BARCELONA, SPAIN	08-Apr-2011
210048	Gine Soca, Eva	CEIC H Clinic i provincial de Barcelona, C/ Villarroel, 170, 8036, Barcelona, BARCELONA, SPAIN	08-Apr-2011
210048	Gine Soca, Eva	CEIC Hospital Vall D'Hebron, PASSEIG DE LA VALL D'HEBRON S/N 119-129, 08035, BARCELONA, BARCELONA, SPAIN	08-Apr-2011

Site #	Investigator	EC/IRB Name and Address	IRB/EC Approval
210095	CANNELL, PAUL	Royal Perth Hospital Ethics Committee, Level 3, Colonial House, Royal Perth Hospital, Wellington Street, 6000, Perth, Western Australia, AUSTRALIA	24-Aug-2011
210096	CATALANO, JOHN	Peninsula Health Research & Ethics Committee, Frankston Hospital, PO Box 52, 3199, Frankston, Victoria, AUSTRALIA	27-Sep-2011
210097	Opat, Stephen	Southern Health Human Research Ethics Committee, 246 CLAYTON ROAD, 3168, CLAYTON, Victoria, AUSTRALIA	21-Jun-2011
210189	VRANOVSKY, ANDREJ	Etická komisia, Národný onkologický ústav, KLENOVA 1, BRATISLAVA, 833 10, BRATISLAVA, SLOVAKIA	15-Jun-2011
210198	TRNENY, MAREK	Eticka Komise Vseobecne fakultni nemocnice, Na Bojisti 1, 128 08, Praha 2, CZECH REPUBLIC	14-Jul-2011
210199	MAYER, JIRI	Fakultni nemocnice Brno; Eticka komise, Jihlavska 20, 625 00, Brno, CZECH REPUBLIC	14-Jul-2011
210200	BELADA, DAVID	Fakultni nemocnice Hradec Kralove; Eticka komise, Sokolska 581, 500 05, Hradec Kralove, CZECH REPUBLIC	14-Jul-2011
210326	SCHNELL, FREDERICK M.	Western Institutional Review Board WIRB Panel 7, 1019 39th Ave S.E., Puyallup, WA, 98374, UNITED STATES	30-Aug-2011
210417	ROSTA, ANDRAS	Medical Research Council, Ethics Committee for Clinical Pharmacology, Arany J. u. 6-8., 1051, Budapest, HUNGARY	05-Sep-2011
210418	Mikala, Gabor	Medical Research Council, Ethics Committee for Clinical Pharmacology, Arany J. u. 6-8., 1051, Budapest, HUNGARY	05-Sep-2011
210419	ILLES, ARPAD	Medical Research Council, Ethics Committee for Clinical Pharmacology, Arany J. u. 6-8., 1051, Budapest, HUNGARY	05-Sep-2011
210420	EGYED, MIKLOS	Medical Research Council, Ethics Committee for Clinical Pharmacology, Arany J. u. 6-8., 1051, Budapest, HUNGARY	05-Sep-2011
210421	DEMETER, JUDIT	Medical Research Council, Ethics Committee for Clinical Pharmacology, Arany J. u. 6-8., 1051, Budapest, HUNGARY	05-Sep-2011
210447	Wrobel, Tomasz	Komisja Bioetyki Uniwersytetu Medycznego w Lodzi, ul. Zeligowskiego 7/9, 90-752, Lodz, POLAND	12-Mar-2013
210448	HELLMANN, ANDRZEJ	Komisja Bioetyki Uniwersytetu Medycznego w Lodzi, ul. Zeligowskiego 7/9, 90-752, Lodz, POLAND	12-Mar-2013
210449	Pluta, Andrzej	Komisja Bioetyki Uniwersytetu Medycznego w Lodzi, ul. Zeligowskiego 7/9, 90-752, Lodz, POLAND	13-Mar-2013
210504	Gomez Almaguer, David	Comite de Etica y Comité de Investigacion de la Fac de Med de la UANL y HospUniv Dr. Jose E Gonzalez, Francisco Ignacio Madero y av. Gonzalitos, s/n col. mitras centro, 64460, Monterrey, NUEVO LEON, MEXICO	13-Feb-2013

Site #	Investigator	EC/IRB Name and Address	IRB/EC Approval
210600	Flinn, Ian	Western Institutional Review Board WIRB Panel 7, 1019 39th Ave S.E., Puyallup, WA, 98374, UNITED STATES	10-Dec-2012
210601	Arrowsmith, Edward	Western Institutional Review Board WIRB Panel 7, 1019 39th Ave S.E., Puyallup, WA, 98374, UNITED STATES	02-Aug-2011
210612	Zallio, Francesco	COMITATO ETICO INTERAZIENDALE AZIENDA OSPEDALIERA DI ALESSANDRIA SS ANTONIO E BIAGIO E CESARE ARRIGO, VIA VENEZIA, 16, 15100, ALESSANDRIA, Piemonte, ITALY	17-Apr-2013
210613	Specchia, Giorgina	IEC Azienda Policlinico Consorziale di Bari, P.zza Giulio Cesare, 11, 70124, Bari, Puglia, ITALY	31-Jul-2013
210614	ROSSI, GIUSEPPE	Comitato Etico della Provincia di Brescia, P.zza Spedali Civili, 1, 25123, Brescia, Lombardia, ITALY	07-Jun-2011
210615	DI RAIMONDO, FRANCESCO	COMITATO ETICO CATANIA 1, VIA S. SOFIA 78, 95123, CATANIA, Sicilia, ITALY	30-May-2011
210616	Congiu, Angela Giovanna	Comitato Etico Regione Liguria (Sezione 2), LARGO ROSANNA BENZI 10, 16132, GENOVA, Liguria, ITALY	23-Jun-2011
210617	Vanazzi, Anna	Comitato Etico Degli IRCCS Istituto Europeo di Oncologia e Centro Cardiologico Monzino, VIA RIPAMONTI 435, 20141, MILANO, Lombardia, ITALY	27-Jul-2011
210620	Califano, Catello	Comitato Etico Campania Sud, Piazza San Giovanni, 80031, Brusciano, Campania, ITALY	21-May-2013
210621	GAIDANO, GIANLUCA	A.O.U. Maggiore della Carità	13-May-2011
210622	PETRINI, MARIO	Comitato Etico Regione Toscana - Area Vasta Nord Ovest, Via Roma 67, c/o Presidio Ospedaliero, 56126, Pisa, Toscana, ITALY	26-May-2011
210624	MERLI, FRANCESCO	Comitato Etico Provinciale Di Reggio Emilia, V.le Risorgimento 57, c/o Direzione Sanitaria, 42100, Reggio Emilia, Emilia-Romagna, ITALY	20-Jun-2011
210625	MARTELLI, MAURIZIO	CE DELL'UNIVERSITA' "SAPIENZA" - POLICLINICO UNIVERSITARIO UMBERTO I - AZIENDA OSPEDALIERA S.ANDREA, Viale Policlinico, 155, 00161, Roma, Lazio, ITALY	21-Jul-2011
210626	Cascavilla, Nicola	COMITATO ETICO DELL'IRCCS GIOVANNI PAOLO II DI BARI PRESSO IRCCS CASA SOLLIEVO DELLA SOFFERENZA, V. le Cappuccini 1, 71013, San Giovanni Rotondo, Puglia, ITALY	06-Sep-2011
210627	LIBERATI, ANNA MARINA	Comitato Etico delle Aziende Sanitarie dell'Umbria, Via Della Rivoluzione, 16, 06073, Ellera Di Corciano, Umbria, ITALY	21-Mar-2013
210628	GHERLINZONI, FILIPPO	Comitato Etico Per la Sperimentazione Clinica (CESC) della Provincia di Treviso e Belluno, Via Sant'Ambrogio di Fiera 37, 31100, Treviso, Veneto, ITALY	27-May-2013

Site #	Investigator	EC/IRB Name and Address	IRB/EC Approval
210629	ZAJA, FRANCESCO	Comitato Etico Unico Regionale, Via Pozzuolo,, 330, 33100, Udine, Friuli-Venezia Giulia, ITALY	20-Sep-2011
210630	KRAMPERA, MAURO	CESC DELLE PROVINCE DI VERONA E ROVIGO, Piazzale A. Stefani, 1, 37126, Verona, Veneto, ITALY	31-Aug-2011
210631	Cavallo, Federica	A.O. Città della salute e della scienza di Torino; Comitato Etico, Corso Bramante 88/90, 10126, Torino, Piemonte, ITALY	13-Jun-2011
210632	BOSI, ALBERTO	COMITATO ETICO AREA VASTA CENTRO, Largo Brambilla 3, 50139, Firenze, Toscana, ITALY	13-Jun-2011
210634	VITOLO, UMBERTO	A.O. Città della salute e della scienza di Torino; Comitato Etico, Corso Bramante 88/90, 10126, Torino, Piemonte, ITALY	13-Jun-2011
230727	Freilone, Roberto	COMITATO ETICO A.O.U. SAN LUIGI GONZAGA DI ORBASSANO, Via Regione Gonzole, 10, 10043, Orbassano, Piemonte, ITALY	12-Jun-2013
230743	Lunin, Scott	Western Institutional Review Board WIRB Panel 7, 1019 39th Ave S.E., Puyallup, WA, 98374, UNITED STATES	11-Jul-2013
230745	Chen, Franklin	Western Institutional Review Board WIRB Panel 7, 1019 39th Ave S.E., Puyallup, WA, 98374, UNITED STATES	12-Jul-2011
230747	Kudrik, Fred J.	Western Institutional Review Board WIRB Panel 7, 1019 39th Ave S.E., Puyallup, WA, 98374, UNITED STATES	25-Jul-2011
230750	Prager, Diane	Western Institutional Review Board WIRB Panel 7, 1019 39th Ave S.E., Puyallup, WA, 98374, UNITED STATES	17-May-2011
230780	Oliveira, Jose	Comitê de Ética em Pesquisa da Casa de Saúde Santa Marcelina, Rua Santa Marcelina, 177, Itaquera, 08270-070, São Paulo, SP, BRAZIL	26-Feb-2014
230789	Chua, Neil	HREBA - Health Research Ethics Board of Alberta - Cancer Committee, c/o Alberta Innovates - Health Solutions, Suite 1500 - 10104, 103 Avenue NW, T5J 0H8, EDMONTON, Alberta, CANADA	29-Jun-2011
230790	McFarlane, Joshua	Western Institutional Review Board WIRB Panel 7, 1019 39th Ave S.E., Puyallup, WA, 98374, UNITED STATES	28-Jul-2011
230792	STEWART, DOUGLAS	HREBA - Health Research Ethics Board of Alberta - Cancer Committee, c/o Alberta Innovates - Health Solutions, Suite 1500 - 10104, 103 Avenue NW, T5J 0H8, EDMONTON, Alberta, CANADA	25-Jul-2011
230919	ZINZANI, Pier Luigi	Comitato Etico Indipendente dell'Azienda Ospedaliero-Universitaria di Bologna, Viale Albertoni, 15, 40138, Bologna, Emilia-Romagna, ITALY	07-Jun-2011
230920	Stelitano, Caterina	Comitato Etico Regionale - Sezione Area Sud, Via Spirito Santo, 24, 89128, Reggio Calabria, Calabria, ITALY	14-Jun-2011
230921	PINTO, ANTONIO	Comitato Etico IRCCS Pascale, VIA M. SEMMOLA 1, 80131, NAPOLI, Campania, ITALY	20-Jun-2011

Site #	Investigator	EC/IRB Name and Address	IRB/EC Approval
230922	Rege Cambrin, Giovanna	COMITATO ETICO A.O.U. SAN LUIGI GONZAGA DI ORBASSANO, Via Regione Gonzole, 10, 10043, Orbassano, Piemonte, ITALY	03-May-2013
230923	Ferreri, Andres	Comitato Etico Irccs Ospedale San Raffaele, VIA OLGETTINA 60, 20132, MILANO, Lombardia, ITALY	14-Jul-2011
231027	KIM, WONSEOG	Samsung Medical Center Institutional Review Board, 81, Irwon-ro, Gangnam-gu, 06351, Seoul, KOREA, REPUBLIC OF	11-Apr-2011
231028	Suh, Cheolwon	Asan Medical Center Ethics Committee, 88, Olympic-ro 43-gil, Songpa-gu, 05505, Seoul, KOREA, REPUBLIC OF	18-Apr-2011
231030	Kim, Jin Seok	SeveranceHospital- YonseiUniversity; IRB, 2F, GwangHye-gwan, 50-1, Yonsei-ro, Seodaemun-gu, 03722, Seoul, KOREA, REPUBLIC OF	15-Apr-2011
231032	Kim, Tae Min	Seoul National University Hospital; IRB, 101, Daehak-ro, Jongno-gu, 03080, Seoul, KOREA, REPUBLIC OF	17-May-2011
231147	Chang, Hung	Chang Gung Med Found, Institutional Review Board, No. 123, Dunghu Rd., Jioulu Village, Taoyuan County, 333, Gueishan Township, TAIWAN	07-Mar-2013
231149	HSIAO, LIANG-TSAI	TVGH Institutional Review Board, No.201, Shih-Pai Road, Sec.2, 112, Taipei, TAIWAN	08-Feb-2013
231348	PAVONE, VINCENZO	Comitato Etico Area 3 - ASL Lecce, Via Miglietta, 5, 73100, LECCE, Puglia, ITALY	26-Mar-2013
231349	Mannina, Donato	Comitato Etico Interaziendale della Provincia di Messina, via Consolare Valeria, 98125, Messina, Sicilia, ITALY	25-Mar-2013
231350	CORRADINI, PAOLO	Comitato Etico Indipendente della Fondazione IRCCS Istituto Nazionale dei Tumori di Milano, Via Giacomo Veneziani 1, 20133, Milano, Lombardia, ITALY	30-Jun-2011
231351	Arcaini, Luca	Comitato Etico Referente per l'Area di Pavia, VIALE GOLGI 19, 27100, PAVIA, Lombardia, ITALY	06-Jun-2011
231509	Zaragoza Vazquez, Alva Nora	Comite de etica en investigacion CECAN Chihuahua; Comite de etica en investigacion CECAN Chihuahua, Ejército Mexicano No. 3700, Col. Centro, C.P.31000., Chihuahua, CHIHUAHUA, MEXICO	20-Nov-2012
231800	CRUMP, MICHAEL	University Health Network Research Ethics Board, 700 University Avenue, 8th Floor, Room 8-19, M5G1Z5, Toronto, Ontario, CANADA	01-Feb-2012
231802	Johnson, Nathalie	McGill University; McGill University; Ethics Board, 3655 Promenade Sir William Osler - 6th Floor, H3G 1Y6, Montreal, Quebec, CANADA	09-Sep-2011
231805	SEHN, LAURIE	UBC BCCA Research Ethics Board (BCCA REB), 600 - 750 West Broadway, Fairmont Medical Building, Room 652, V5Z 1H5, Vancouver, British Columbia, CANADA	20-Feb-2012

Site #	Investigator	EC/IRB Name and Address	IRB/EC Approval
231806	Keating, Mary-Margaret	Nova Scotia Health Authority Research Ethics Board, QEII Health Science Centre for Clinical Res, Room 118-5790 University Avenue, B3H 1V7, Halifax, Nova Scotia, CANADA	12-Mar-2012
231810	Larouche, Jean-Francois	Comité d'éthique de la recherche du CHU de Québec, 10 rue de L'Espinay, Suite A0-124, G1L 3L5, Québec, Quebec, CANADA	07-Sep-2011
231855	WRIGHT, DAVID	Western Institutional Review Board WIRB Panel 7, 1019 39th Ave S.E., Puyallup, WA, 98374, UNITED STATES	27-Jun-2013
231888	Ramirez, Carlos	Comite De Etica En Investigacion Fundacion Universitaria Sanitas, Carrera 7 # 173 - 64, Bogota, COLOMBIA	15-Mar-2012
231917	Elemary, Mohamed	University of Saskatchewan Biomedical Research Ethics Board, 117 Science Place, Room 305 Kirk Hall, S7N 5C8, Saskatoon, Saskatchewan, CANADA	22-Jun-2011
232136	Ramirez Romero, Eva Fabiola	OSMO, S.C.; Comité de ética en investigación y comité de investigación, Himboldt 302, col. centro, C.P. 68000, Oaxaca, OAXACA, MEXICO	28-Jan-2013
235676	Terui, Yasuhito	The Cancer Institute Hospital of JFCR Institutional Review Board, 3-8-31 Ariake Koto-Ku, 135-8550, Tokyo, JAPAN	07-Sep-2011
235677	Maruyama, Dai	National Cancer Center Institutional Review Board, 5-1-1 Tsukiji Chuo-Ku, 104-0045, Tokyo, JAPAN	01-Nov-2011
235679	Uchida, Toshiki	Japanese Red Cross Nagoya Daini Hospital Institutional Review Board, 2-9 Myoken-cho Showa-Ku Nagoya-shi, 466-8650, Aichi, JAPAN	28-Sep-2011
235680	Yamamoto, Go	Toranomon Hospital and Toranomon Hospital Kajigaya Institutional Review Board, 2-2-2 Toranomon, Minato-ku, 105-8470, Tokyo, JAPAN	17-Oct-2011
236213	Kanakura, Yuzuru	Osaka University Hospital Institutional Review Board, 2-15, Yamadaoka, Suita-shi, 565-0871, Osaka, JAPAN	11-Nov-2011
238086	Mollica, Luigina	Comité d'éthique de la recherche HMR, Pavillion Rachel-Touriguy, 5415 boul de L'Assomption, 4th floor, H1T 2M4, Montreal, Quebec, CANADA	14-Dec-2011
238173	BENCE-BRUCKLER, ISABELLE	Ottawa Hospital Research Ethics Board, Civic Box 411, 726 Parkdale Avenue, K1Y 4E9, Ottawa, Ontario, CANADA	24-Jan-2012
245517	Kroschinsky, Frank	EK Würzburg, Versbacher Str. 9, 97078, Würzburg, GERMANY	30-Aug-2012
245517	Kroschinsky, Frank	Ethik-Kommission am Universitätsklinikum Carl-Gustav-Carus Technische Universität Dresden, Fetscherstrasse 74, 01307, Dresden, GERMANY	30-Aug-2012
245519	Witzens-Harig, Mathias	EK Würzburg, Versbacher Str. 9, 97078, Würzburg, GERMANY	30-Aug-2012

Site #	Investigator	EC/IRB Name and Address	IRB/EC Approval
245519	Witzens-Harig, Mathias	Ethikkommission der Medizinischen Fakultät Heidelberg, Alte Glockengießerei 11/1, 69115, Heidelberg, GERMANY	30-Aug-2012
245597	Topp, Max	EK Würzburg, Versbacher Str. 9, 97078, Würzburg, GERMANY	30-Aug-2012
245597	Topp, Max	Ethik-Kommission der Medizinischen Fakultät der Friedrich-Alexander-Universität Erlangen-Nürnberg, Krankenhausstr. 12, 91054, Erlangen, GERMANY	30-Aug-2012
245598	Krause, Stefan	EK Würzburg, Versbacher Str. 9, 97078, Würzburg, GERMANY	30-Aug-2012
245598	Krause, Stefan	Ethik-Kommission der Medizinischen Fakultät der Friedrich-Alexander-Universität Erlangen-Nürnberg, Krankenhausstr. 12, 91054, Erlangen, GERMANY	30-Aug-2012
245599	Kurz-zur Hausen, Anna Kordelia	EK Würzburg, Versbacher Str. 9, 97078, Würzburg, GERMANY	30-Aug-2012
247997	Basso, Alfredo	Comite de Docencia e Investigacion del Sanatorio Parque, Boulevard Oroño 860, 2000, Rosario, ARGENTINA	09-Nov-2012
247997	Basso, Alfredo	Comite Indep. De Etica Para Ensayos En Farmacolog., Pte. J. E. Uriburu 774, 1er Piso, C1027AAP, Buenos Aires, ARGENTINA	09-Nov-2012
250709	Tatsumi, Yoichi	Kindai University Hospital Institutional Review Board, 377-2 Ohnohigashi, Osaka-Sayama-shi, 589-8511, Osaka, JAPAN	11-Sep-2012
250710	Akashi, Koichi	Kyushu University Hospital IRB, 3-1-1 Maidashi, Higashi-Ku, Fukuoka-Shi, 812-8582, Fukuoka, JAPAN	05-Oct-2012
250711	Nakamae, Hirohisa	Osaka City University Hospital IRB, 1-5-7,Asahimachi, Abeno-ku, Osaka-shi, 545-8586, Osaka, JAPAN	31-Oct-2012
251201	Ueda, Yasunori	Kurashiki Central Hospital Institutional Review Board, 1-1-1 Miwa, Kurashiki-shi, 710-8602, Okayama, JAPAN	15-Oct-2012
251406	Hamed, Aryan	Medical Research Council, Ethics Committee for Clinical Pharmacology, Arany J. u. 6-8., 1051, Budapest, HUNGARY	26-Sep-2012
251624	McDonald, Andrew	Pharma-Ethics Independent Research Ethics Committee, 123 Amcor Road, Lyttleton, 0157, Centurion, SOUTH AFRICA	22-Nov-2012
251626	COHEN, GRAHAM	Pharma-Ethics Independent Research Ethics Committee, 123 Amcor Road, Lyttelton, 0157, Centurion, SOUTH AFRICA	22-Nov-2012
251628	RAPOPORT, BERNARDO	Pharma-Ethics Independent Research Ethics Committee, 123 Amcor Road, Lyttelton, 0157, Centurion, SOUTH AFRICA	22-Nov-2012
251630	SAVIC, IVANKA	Ethics Committee Clinical Center Vojvodine, Hajduk Veljkova 1, 21000, Novi Sad, SERBIA	10-Dec-2012
251631	MIHALJEVIC, BILJANA	Ethics Committee Clinical Center Of Serbia, PASTEROVA 2, 11000, BELGRADE, SERBIA	13-Dec-2012

Site #	Investigator	EC/IRB Name and Address	IRB/EC Approval
251856	ROBAK, TADEUSZ	Komisja Bioetyki Uniwersytetu Medycznego w Lodzi, ul. Zeligowskiego 7/9, 90-752, Lodz, POLAND	13-Mar-2013
251857	WALEWSKI, JAN	Komisja Bioetyki Uniwersytetu Medycznego w Lodzi, ul. Zeligowskiego 7/9, 90-752, Lodz, POLAND	13-Mar-2013
252760	Tamura, Hideto	Nippon Medical School Hospital Institutional Review Board, 1-1-5 Sendagi, Bunkyo-ku, 113-8603, Tokyo, JAPAN	25-Oct-2012
252864	Chiou, Lun-Wei	Koo Fundation Sun Yat-Sen Cancer Center, IRB, 125 Lih-Der Road, Pei-Tou District, 112, Taipei, TAIWAN	10-Apr-2013
253012	ZHU, JUN	The Ethics Committee of Beijing Cancer Hospital, No.52 Fucheng Road,, Haidian District,, 100036, Beijing, CHINA	02-Apr-2013
253013	Zhang, Qingyuan	EC of The Cancer Hospital of Harbin Medical University; Ethics Committee, Rm609, Admin building, No. 150 Ha Ping Road, 150081, Harbin, CHINA	29-Jan-2013
253023	Du, Xin	EC of Guangdong General Hospital, 106, ZHONGSHAN ER ROAD, 510080, GUANGZHOU, CHINA	09-Jun-2013
253024	WU, GANG	Ethics Committee of Tongji Medical College, HUST, 1095 Jie Fang Da Dao Rd., Wuhan, Hu Bei Province, 430030, Wuhan, CHINA	28-Jan-2013
253172	Ren, Hanyun	The Ethics Committee of Peking University First Hospital, No.6 Dahongluochang Street, Xicheng District, 100034, Beijing, CHINA	27-Mar-2013
253174	Li, Wei	the First Hospital of Jilin University; The Committee of The first Hospital of Jilin University, 71 Xinmin Street, Changchun City, Jilin Province, 130021, Changchun, CHINA	07-Feb-2013
253176	Xiao, Xiubin	307 Hospital Ethics Committee, Number 8, Fengtai east street, 100071, Beijing, CHINA	01-Mar-2013
253177	SHI, YUAN KAI	Local EC of CHINESE ACADEMY OF MEDICAL SCIENCE; CANCER INST. & HOSPITAL, Number17, Panjiayuan south road, 100021, Beijng, CHINA	01-Apr-2013
253199	Liu, Li	EC of Tangdu Hospital, NO. 1,Xin Si Road,Baqiao District, Xi'an City,Shannxi Province, 710038, Xi`an, CHINA	26-Feb-2013
253204	Zhang, Huilai	EC of Tianjin Cancer Hospital, Ti-Yuan-Bei, Huan-Hu-Xi Road, He X1 District, 300060, Tianjin, CHINA	12-Apr-2013
253213	Hong, Xiaonan	EC of Fudan University Shanghai Cancer Center, 5th Floor, Building 2, No.270, Dong'an Road, 200032, Shanghai, CHINA	28-Jan-2013
253214	Jin, Jie	1st Affliated Hospital Of Zhejiang University, No.79 Qingchun Road, 310009, Hangzhou, CHINA	21-May-2013
253220	Yang, Yu	EC of Fujian Cancer Hospital, No. 91, Fuma Road, 350014, Fuzhou, CHINA	27-Mar-2013
253230	Hu, Jianda	EC of Fujian Medical University Union Hospital, No.29 Xinquan Road, 350001, Fuzhou, CHINA	09-Apr-2013

Site #	Investigator	EC/IRB Name and Address	IRB/EC Approval
253231	FENG, JIFENG	JiangSu Province Cancer Hospital/Ethic Committee Office, No. 42 Bai Zi Ting Road, 210009, Nanjing, CHINA	30-Jan-2013
253232	Li, Junmin	EC of RUIJIN HOSPITAL, No.197,The second Ruijin Road, Huangpu District,Shanghai, 200025, Shanghai, CHINA	01-Apr-2013
253233	WANG, JIANMIN	Shanghai Changhai Hospital, Room 212, Building 17, 174 Changhai Road, 200433, Shanghai, CHINA	26-Feb-2013
253234	WU, Depei	EC of First Affiliated Hospital of Soochow University, 96 SHIZI STREET, 215006, SUZHOU, CHINA	19-Mar-2013
253423	Lai, Yongrong	Ethics Committee of The First Hospital of Guangxi Medical University, No. 6 Shuang Yang Road, 530021, Nanning, CHINA	06-Feb-2013
253425	Li, Jianyong	EC of Jiangsu Province People Hosptial, NO 300 GUANGZHOU RD, 210029, NANJING, CHINA	28-Jan-2013
253429	Li, Yan	EC of The First Hospital of China Medical University, No. 155, Nanjingbei Road, Heping District, Shenyang, 110001, Shenyang, CHINA	27-Jun-2013
253431	Huang, Huiqiang	EC of Sun Yet-sen University Cancer Center, No. 651, Dongfeng East Road, 510060, Guangzhou, CHINA	30-Jan-2013
253781	Segovia, Javier	Comité de Ética en Investigación Clínica, Calle 163A No 13B-60, Bogota, COLOMBIA	06-Mar-2013
253844	Lemieux, Bernard	Comité d'éthique de la recherche, CHUM, Pavillion R, 900 Rue St-Denis, 3rd Floor, H2X 0A9, Montreal, Quebec, CANADA	18-Apr-2013
254286	Gonzalez Lopez Elizalde, Montserrat	Comite de Investigacion CECLIQ; Comite de Investigacion CECLIQ, Prol. Privada Ignacio Zaragoza,, No. 16-B 2do piso, Col. Centro, C.P. 76000, Querétaro, Qro., Queretaro, QUERETARO, MEXICO	09-Jan-2013
254300	Yi, Pingyong	EC of Hunan Provincial Cancer Hospital, 283 Tongzipo Raod, Yuelu District, 410006, Changsha, CHINA	02-Apr-2013
254304	Hu, Yu	Ethics Committee of Tongji Med. Col, Huazhong Univ. of Sci. & Tech, No.1277 Jiefang Dadao, 430022, Wuhan City, CHINA	28-Jan-2013
254888	Valdiviezo, Natalia	Comité Institucional de Etica en Investigación de la Asociación Benéfica Prisma, Carlos Gonzales #251 Urbanización Maranga San Miguel, Lima 32, Lima, PERU	04-Feb-2013
254889	Quintana, Shirley	Comité Institucional de Ética en Investigación del INEN, Avenida Angamos Este 2520, Lima 11, Lima, PERU	04-Mar-2013
254926	Tumyan, Gayane	Blokhin Russian Cancer Research Center Ethics Committee, Kashirskoye shosse,24, Moscow, RUSSIAN FEDERATION	30-Jul-2013
254933	Chagorova, Tatiana	E.C. at the Penza Regional Oncology Dispensary, 37A, prospekt Stroiteley, 440071, Penza, RUSSIAN FEDERATION	08-Feb-2013

Site #	Investigator	EC/IRB Name and Address	IRB/EC Approval
254934	Kuzmin, Alexey	Republican Clinical Oncology Dispensary of MoH of Tatarstan, 29, Sibirskiy trakt, 420029, Kazan, RUSSIAN FEDERATION	15-Mar-2013
254939	SAMOYLOVA, OLGA	E.C.at the Nizhny Novgorod Regional Clinical Hospital N.A. Semashko, 190, Rodionova street, 306126, Nizhny Novgorod, RUSSIAN FEDERATION	27-Dec-2012
254942	MYASNIKOV, ALEXANDR	E.C. of Republican Hospital Named After V.A. Baranov, Pirogova street, 3, 185019, Petrozavodsk, RUSSIAN FEDERATION	25-Jan-2013
255301	Cho, Seok Goo	Seoul St. Mary's Hospital; IRB, 222, Banpo-daero, Seocho-gu, 06591, Seoul, KOREA, REPUBLIC OF	07-Feb-2013
255302	Eom, Hyeon Seok	IRB of National Cancer Center, 323, Ilsan-ro, Ilsandong-gu, Goyang-si, 10408, Gyeonggi-do, KOREA, REPUBLIC OF	15-Jan-2013
255303	Lee, Je-Jung	Chonnam National University Hwasun Hospital; IRB, 322, Seoyang-ro, Hwasun-eup, Hwasun-gun, 58128, Jeollanam-do, KOREA, REPUBLIC OF	26-Dec-2012
255348	Shi, Qingzhi	EC of the 2nd Affiliated Hospital of Nanchang University, No.1 Minde Road, 330008, Nanchang, CHINA	30-Jan-2013
255971	Fu, Jinxiang	EC of Second Affiliated Hospital of Soochow University, 1055 Sanxiang Road, 215004, Suzhou, CHINA	07-Feb-2013
256257	ROITMAN, DARYL	Ontario Cancer Research Ethics Board, MaRS Centre, South Tower, 101 College Street, Suite 500, M5G 1L7, Toronto, Ontario, CANADA	11-Mar-2013
257526	Nagafuji, Koji	Kurume University Institutional Review Board, 67 Asahimachi, Kurume-shi, 830-0011, Fukuoka, JAPAN	17-Apr-2013
257527	Nakajima, Hideaki	Yokohama City University Hospital Institutional Review Board, 3-9 Fukuura,Kanazawa-ku,Yokohama-shi, 236-0004, Kanagawa, JAPAN	28-Mar-2013
257528	Nakamura, Nobuhiko	Gifu University Hospital IRB, 1-1 Yanagido, Gifu-shi, 501-1194, Gifu, JAPAN	15-Feb-2013
257579	VISCO, CARLO	Ce Per La Sperim.E Clinica Della Prov. Di Vicenza, c/o Direzione Medica, Via Rodolfi 37, 36100, Vicenza, Veneto, ITALY	06-May-2013
257639	Oyake, Tatsuo	Iwate Medical University Institutional Review Board, 1-1-1, Idai-dori, Yahaba-cho, Shiwa-gun, 028-3600, Iwate, JAPAN	22-Mar-2013
258286	Voloshin, Sergey	EC of Rus SPb Res Inst of Haemot and Transfusiol, 2-ND SOVETSKAYA STREET, 16, 191024, ST. PETERSBURG, RUSSIAN FEDERATION	22-Apr-2013
258642	Teshima, Takanori	Hokkaido University Hospital Institutional Review Board, Kita14-jo,Nishi5-chome,Kita-ku,Sapporo, 060-8648, Hokkaido, JAPAN	12-Mar-2013
258643	Sakaida, Emiko	Chiba University Hospital Institutional Review Board, 1-8-1 Inohana, Chuo-ku, 260-8670, Chiba, JAPAN	18-Mar-2013

Site #	Investigator	EC/IRB Name and Address	IRB/EC Approval
258644	Nishikori, Momoko	Kyoto University Hospital Institutional Review Board, 54 Kawahara-cho Shogoin Sakyo-ku, Kyoto-shi, 606-8507, Kyoto, JAPAN	14-May-2013
258776	Di Simone, Christopher	US Oncology, Inc Institutional Review Board, 10101 Woodloch Forest, The Woodlands, TX, 77380, UNITED STATES	19-Apr-2013
258777	Larson, Timothy	US Oncology, Inc Institutional Review Board, 10101 Woodloch Forest, The Woodlands, TX, 77380, UNITED STATES	19-Apr-2013
258778	GARBO, LAWRENCE	US Oncology, Inc Institutional Review Board, 10101 Woodloch Forest, The Woodlands, TX, 77380, UNITED STATES	19-Apr-2013
258779	Garrison, Mitchell	US Oncology, Inc Institutional Review Board, 10101 Woodloch Forest, The Woodlands, TX, 77380, UNITED STATES	19-Apr-2013
258780	Houck, William	US Oncology, Inc Institutional Review Board, 10101 Woodloch Forest, The Woodlands, TX, 77380, UNITED STATES	19-Apr-2013
258782	KLEIN, LEONARD	US Oncology, Inc Institutional Review Board, 10101 Woodloch Forest, The Woodlands, TX, 77380, UNITED STATES	19-Apr-2013
258785	Zweibach, Alexander	US Oncology, Inc Institutional Review Board, 10101 Woodloch Forest, The Woodlands, TX, 77380, UNITED STATES	19-Apr-2013
258787	Forero, Andres	Western Institutional Review Board WIRB Panel 7, 1019 39th Ave S.E., Puyallup, WA, 98374, UNITED STATES	18-Sep-2013
258788	Fayad, Luis	MD Anderson Cancer Center Office of Protocol Research, 1515 Holcombe Blvd., Box 574, Houston, TX, 77030, UNITED STATES	30-May-2013
258790	Bessudo, Alberto	Western Institutional Review Board WIRB Panel 7, 1019 39th Ave S.E., Puyallup, WA, 98374, UNITED STATES	14-Jun-2013
258791	Sharman, Jeff-Porter	US Oncology, Inc Institutional Review Board, 10101 Woodloch Forest, The Woodlands, TX, 77380, UNITED STATES	19-Apr-2013
259141	Stuart, Robert	Western Institutional Review Board WIRB Panel 7, 1019 39th Ave S.E., Puyallup, WA, 98374, UNITED STATES	30-Sep-2013
259727	Burke, John M.	US Oncology, Inc Institutional Review Board, 10101 Woodloch Forest, The Woodlands, TX, 77380, UNITED STATES	19-Apr-2013
259728	Grosse-Perdekamp, Maria	Western Institutional Review Board WIRB Panel 7, 1019 39th Ave S.E., Puyallup, WA, 98374, UNITED STATES	19-Apr-2013
259729	Goldschmidt-Jr, Jerome	US Oncology, Inc Institutional Review Board, 10101 Woodloch Forest, The Woodlands, TX, 77380, UNITED STATES	19-Apr-2013

Site #	Investigator	EC/IRB Name and Address	IRB/EC Approval
259729	Goldschmidt-Jr, Jerome	US Oncology, Inc. Institutional Review Board, 4144 N. Central Expressway, Suite 1250, Dallas, TX, 75204, UNITED STATES	19-Apr-2013
260470	Johnson, Nathalie	McGill University; McGill University; Ethics Board, 3655 Promenade Sir William Osler - 6th Floor, H3G 1Y6, Montreal, Quebec, CANADA	06-May-2013
261854	PILNIK, NORMA	Comité Independiente de Etica Fundación Rusculleda, Avenida Colon 2057, X5003DCE, Cordoba, ARGENTINA	15-Aug-2013
262115	Cerana Helvecia, Susana	Comite de Etica; del Sanatorio Britanico, Paraguay 40, S2000CVB, Rosario, ARGENTINA	25-Jun-2013
264726	Chou, Takaaki	Niigata Cancer Center Hospital Institutional Review Board, 2-15-3 Kawagishi-cho, Chuo-ku, Niigata-shi, 951-8566, Niigata, JAPAN	11-Oct-2013
264727	Ohmine, Ken	Jichi Medical University Institutional Review Board, 3311-1 Yakushiji, Shimotsuke-shi, 329-0498, Tochigi, JAPAN	31-Oct-2013
264775	Suzumiya, Junji	Shimane University Hospital Institutional Review Board, 89-1 ENYA-CHO IZUMO-SHI, 693-8501, SHIMANE, JAPAN	10-Oct-2013
265383	CASANOVA, LUIS	Comité Institucional de Etica en Investigación de la Asociación Benéfica Prisma, Carlos Gonzales #251 Urbanización Maranga San Miguel, Lima 32, Lima, PERU	10-Oct-2013

Supplementary Figure S1. NMF-defined clusters in CAVALLI and their association with cell-of-origin.

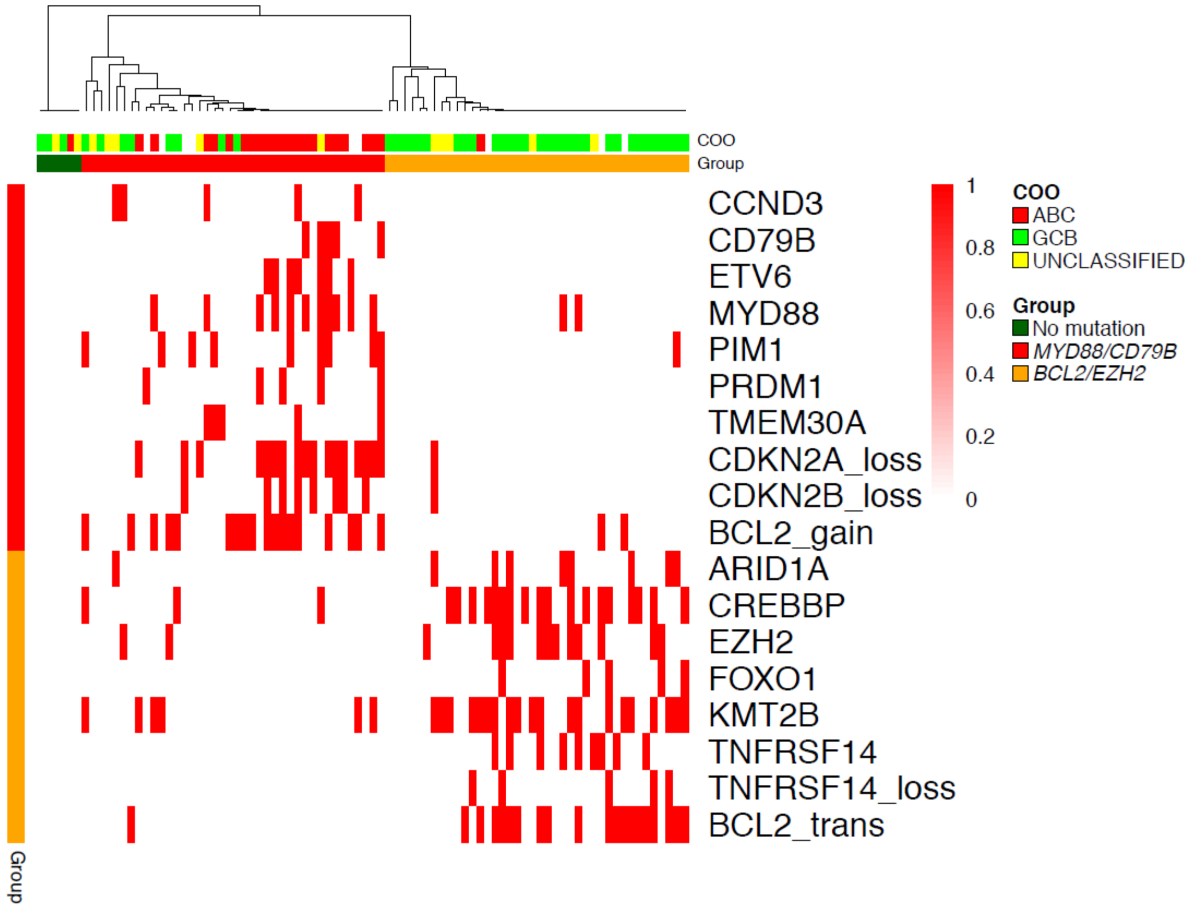

ABC, activated B-cell-like; COO, cell-of-origin; GCB, germinal center B-cell-like; NMF, non-negative matrix factorization.

Supplementary Figure S2. Kaplan-Meier curves of PFS for NMF high- and low-risk prognostic groups in GOYA according to (A) treatment and (B) cell-of-origin (activated B-cell-like vs germinal center B-cell-like).

A

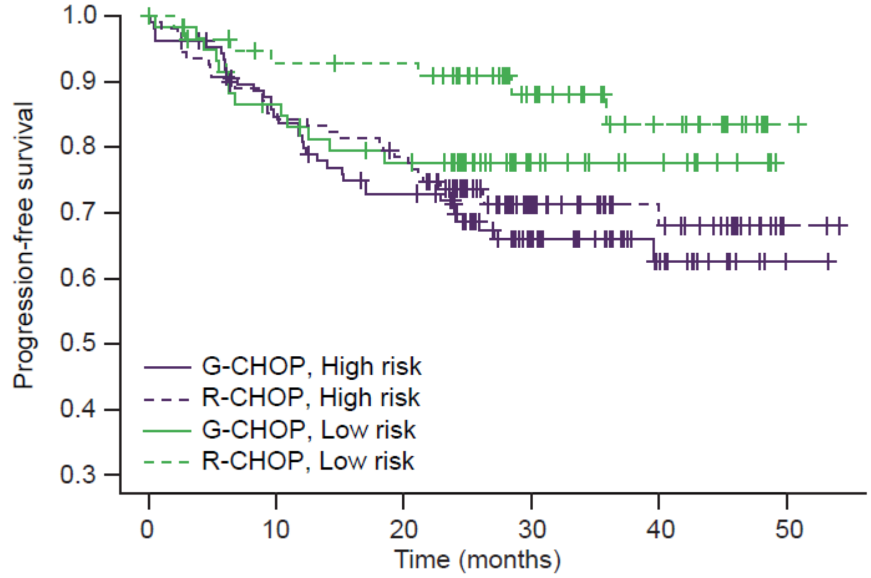

No. at risk						
G-CHOP, High risk	111	87	73	37	16	1
R-CHOP, High risk	111	90	82	42	22	2
G-CHOP, Low risk	62	50	43	18	11	0
R-CHOP, Low risk	59	50	49	29	15	1

B

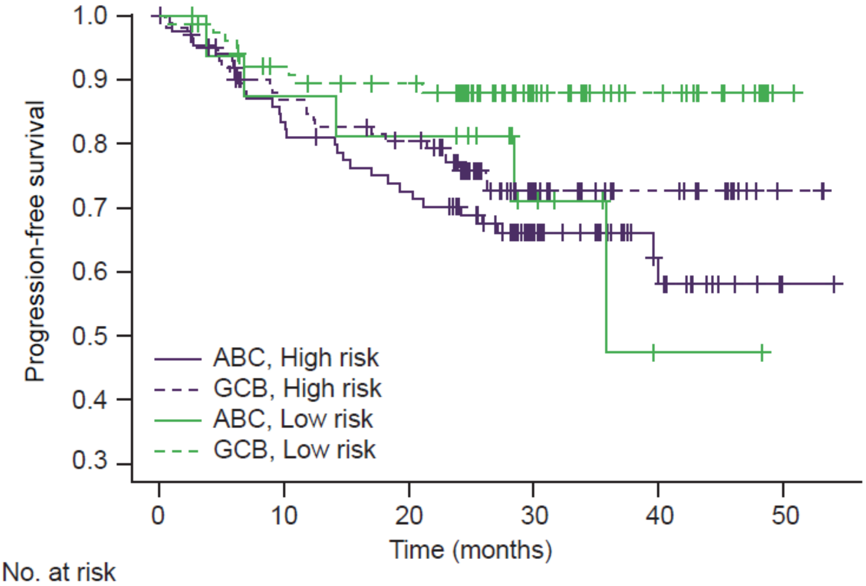

No. at risk						
ABC, High risk	87	70	60	33	15	1
GCB, High risk	104	82	74	33	17	2
ABC, Low risk	18	14	13	6	1	0
GCB, Low risk	78	67	62	33	19	1

ABC, activated B-cell-like; G-CHOP, obinutuzumab plus cyclophosphamide, doxorubicin, vincristine and prednisone; GCB, germinal center B-cell-like; NMF, non-negative matrix factorization; PFS, progression-free survival; R-CHOP, rituximab plus cyclophosphamide, doxorubicin, vincristine and prednisone.

Supplementary Figure S3. Kaplan-Meier curve of PFS for the *BCL2/EZH2* versus *MYD88/CD798* clusters in CAVALLI (*de novo* NMF clustering).

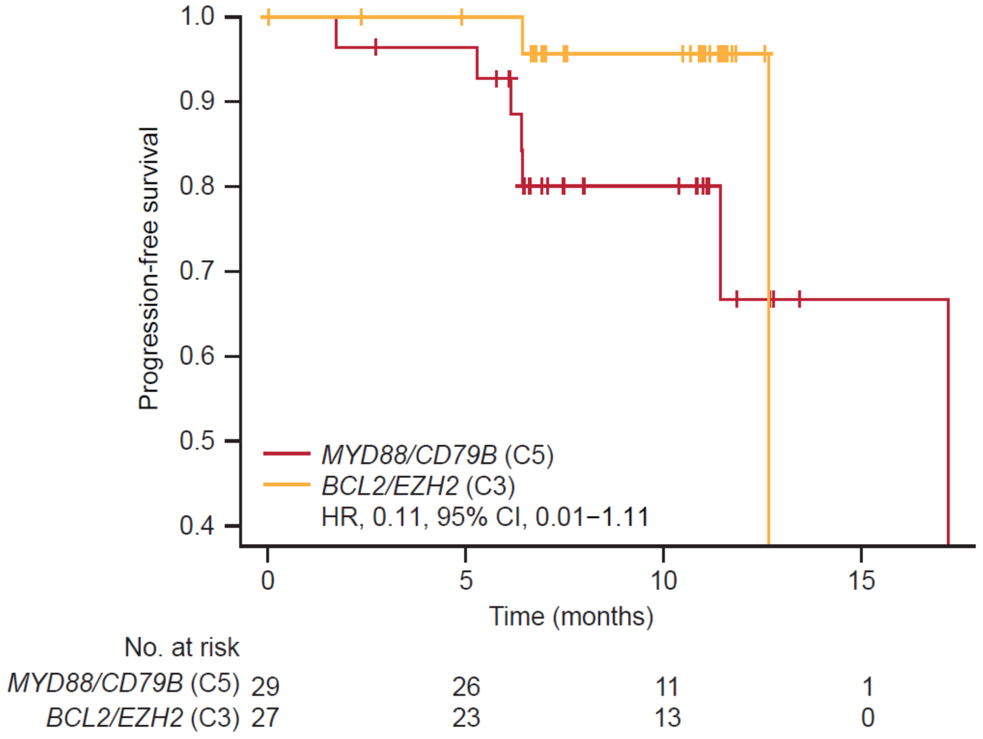

CI, confidence interval; HR, hazard ratio; NMF, non-negative matrix factorization; PFS, progression-free survival.

Supplementary Figure S4. Training of a Random Forest model on NMF cluster labels in GOYA onto CAVALLI. (A) Clustering of gene features of predicted GOYA NMF groups onto CAVALLI. (B) Kaplan-Meier curves of PFS for predicted GOYA NMF groups in CAVALLI.

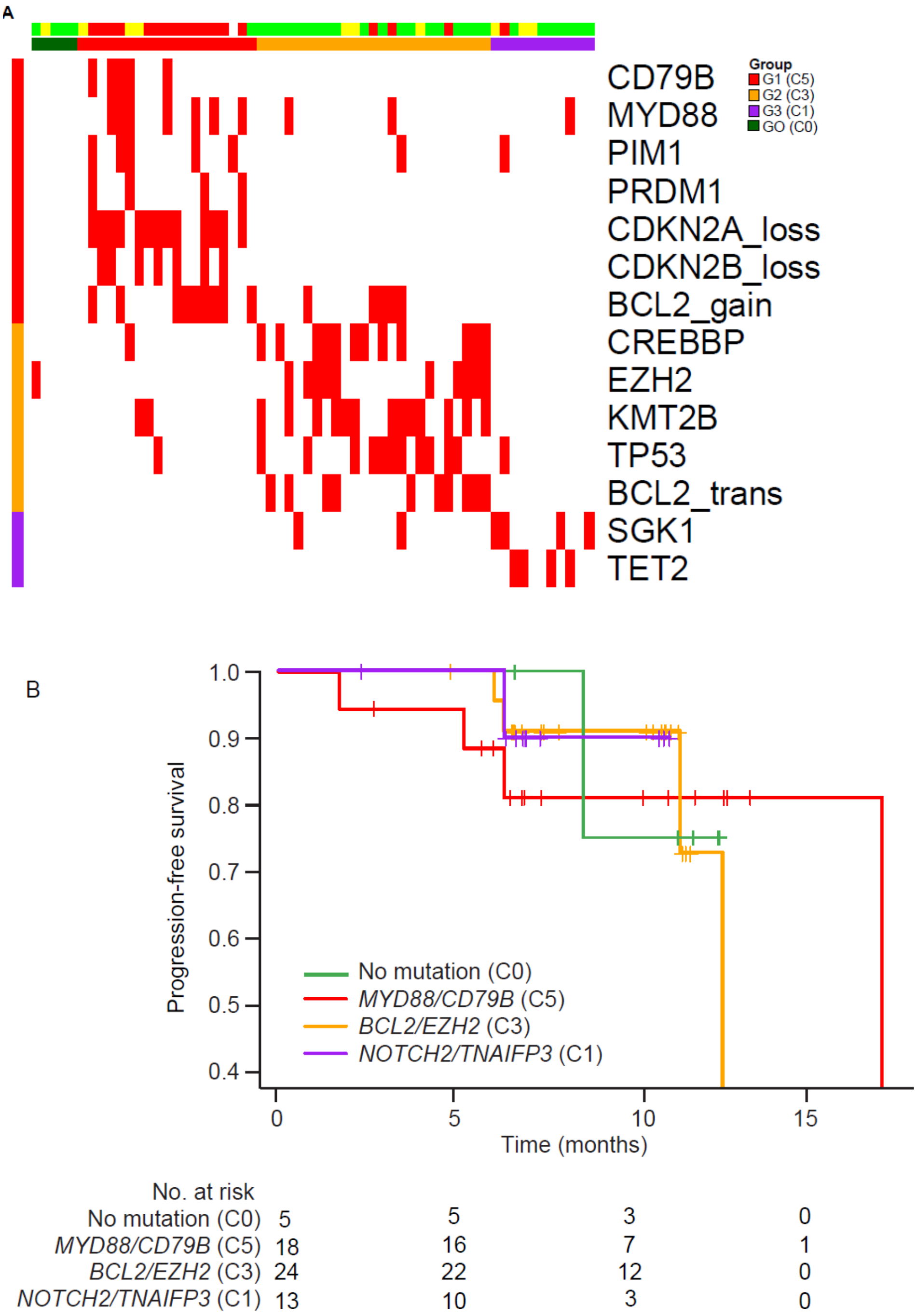

NMF, non-negative matrix factorization; PFS, progression-free survival.

Supplementary Figure S5. Kaplan-Meier curves of PFS for GOYA and CAVALLI

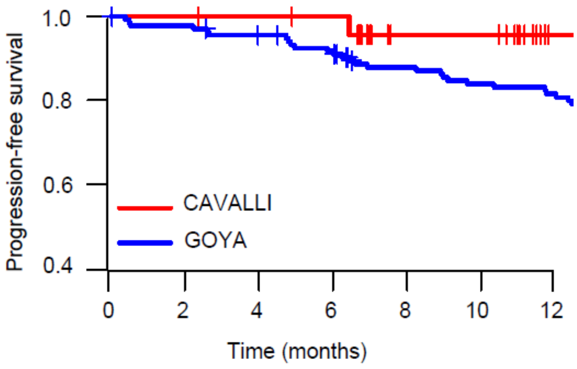

No. at risk							
CAVALLI	27	25	24	23	13	13	2
GOYA	141	132	127	121	111	106	103

PFS, progression-free survival.

Supplementary Figure S6. Differential expression and pathway analysis of NMF high-risk vs low-risk patients in GOYA. (A) Volcano plot of all genes for patients with NMF risk categorization and RNA-Seq data. Limma was used to determine differential expression with no covariate adjustment. Labelled points outside of the red dotted lines indicate genes with a false discovery rate <0.05 (Benjamini-Hochberg) and log-fold change >1. (B) Pathways with false discovery rate <0.05 using fast gene set enrichment analysis. tStat values from Limma were used along with the mSigDB Hallmark signature list. (C) Top 30 ranked normalized enrichment scores for Staudt Signature database. All signatures have adjusted p-values with a false discovery rate <0.05. (D) Additional signature sets from MSigDB.

A

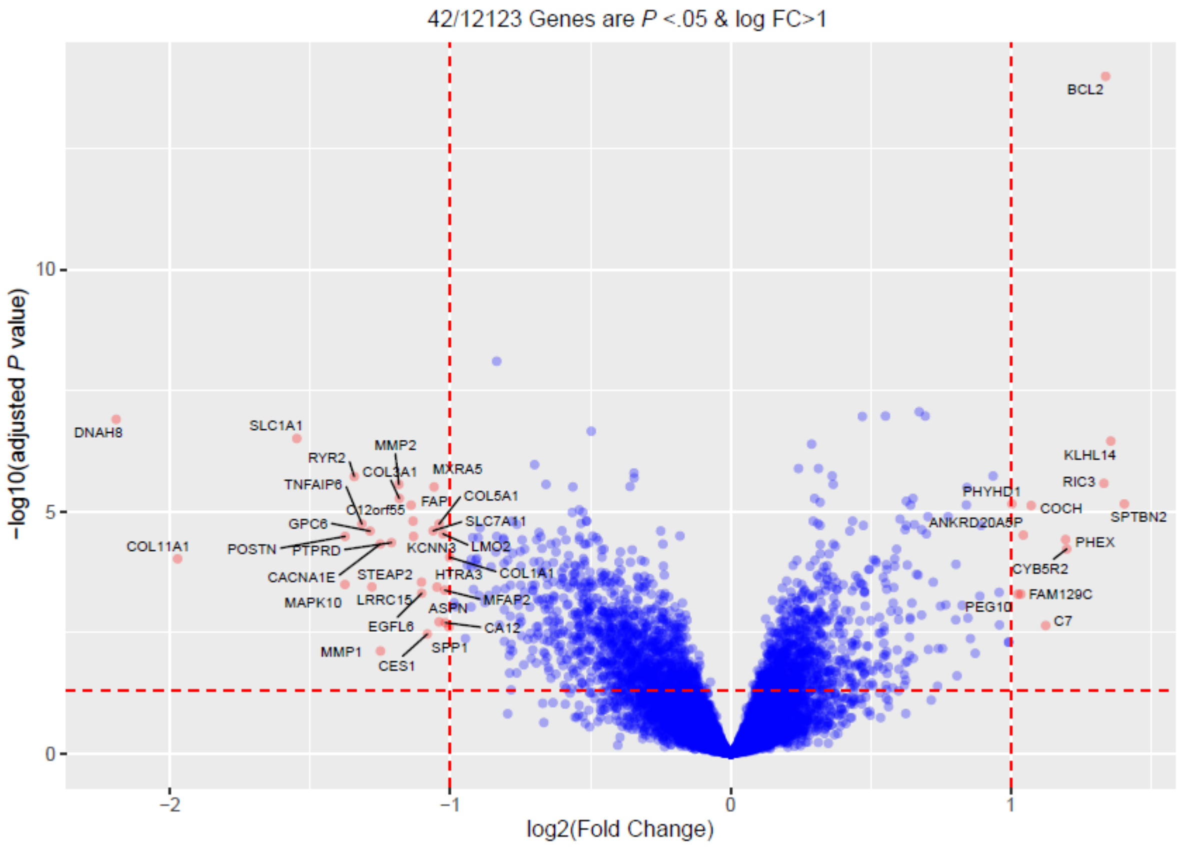

B

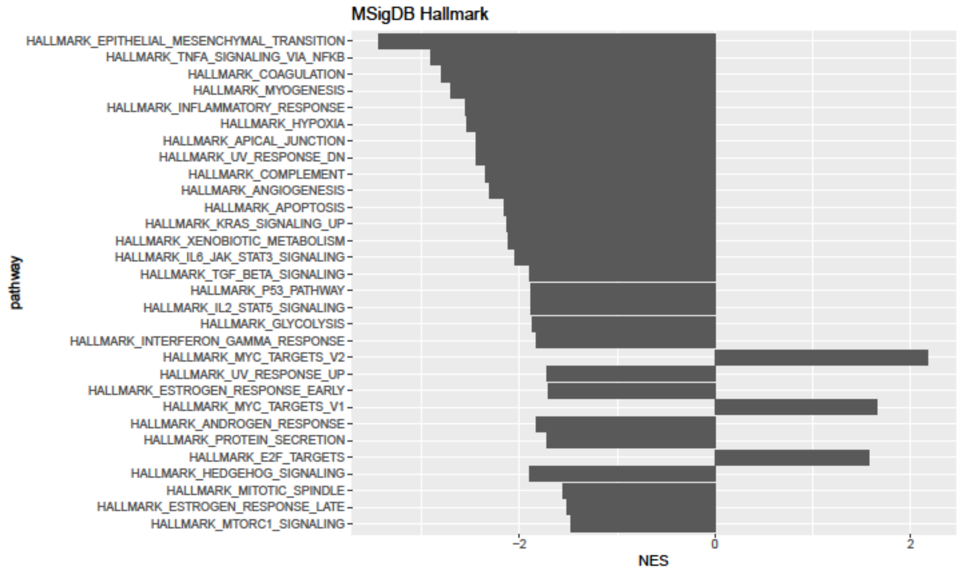
MSigDB Hallmark
HALLMARK_EPITHELIAL_MESENCHYMAL_TRANSITION
HALLMARK_TNFA_SIGNALING_VIA_NFKB
HALLMARK_COAGULATION
HALLMARK_MYOGENESIS
HALLMARK_INFLAMMATORY_RESPONSE
HALLMARK_HYPOXIA
HALLMARK_APICAL_JUNCTION
HALLMARK_UV_RESPONSE_DN
HALLMARK_COMPLEMENT
HALLMARK_ANGIOGENESIS
HALLMARK_APOPTOSIS
HALLMARK_KRAS_SIGNALING_UP
HALLMARK_XENOBIOTIC_METABOLISM
HALLMARK_IL6_JAK_STAT3_SIGNALING
HALLMARK_TGF_BETA_SIGNALING
HALLMARK_P53_PATHWAY
HALLMARK_IL2_STAT5_SIGNALING
HALLMARK_GLYCOLYSIS
HALLMARK_INTERFERON_GAMMA_RESPONSE
HALLMARK_MYC_TARGETS_V2
HALLMARK_UV_RESPONSE_UP
HALLMARK_ESTROGEN_RESPONSE_EARLY
HALLMARK_MYC_TARGETS_V1
HALLMARK_ANDROGEN_RESPONSE
HALLMARK_PROTEIN_SECRETION
HALLMARK_E2F_TARGETS
HALLMARK_HEDGEHOG_SIGNALING
HALLMARK_MITOTIC_SPINDLE
HALLMARK_ESTROGEN_RESPONSE_LATE
HALLMARK_MTORC1_SIGNALING
pathway
−2
0
2
NES

C

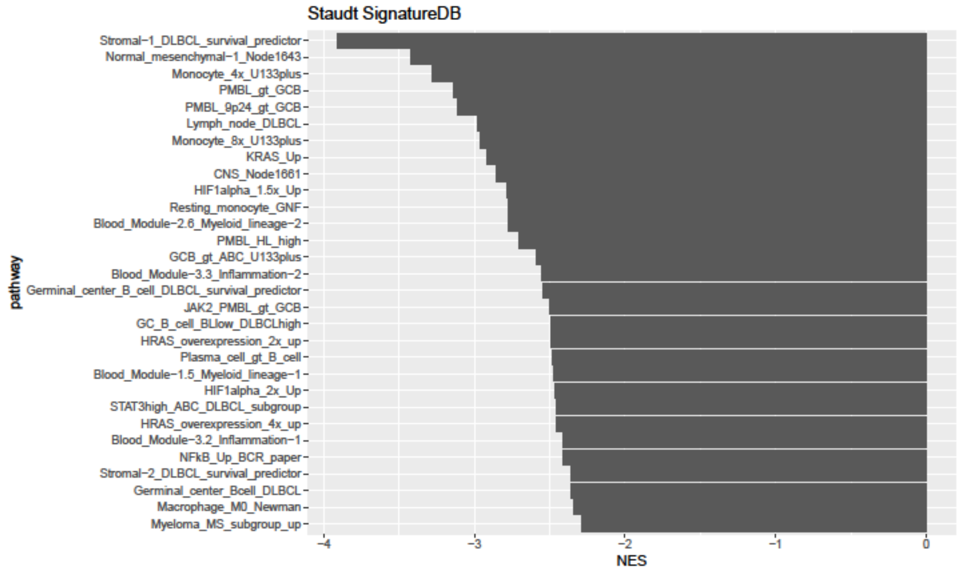
Staudt SignatureDB
Stromal−1_DLBCL_survival_predictor
Normal_mesenchymal−1_Node1643
Monocyte_4x_U133plus
PMBL_gt_GCB
PMBL_9p24_gt_GCB
Lymph_node_DLBCL
Monocyte_8x_U133plus
KRAS_Up
CNS_Node1661
HIF1alpha_1.5x_Up
Resting_monocyte_GNF
Blood_Module−2.6_Myeloid_lineage−2
PMBL_HL_high
GCB_gt_ABC_U133plus
Blood_Module−3.3_Inflammation−2
Germinal_center_B_cell_DLBCL_survival_predictor
JAK2_PMBL_gt_GCB
GC_B_cell_BLlow_DLBCLhigh
HRAS_overexpression_2x_up
Plasma_cell_gt_B_cell
Blood_Module−1.5_Myeloid_lineage−1
HIF1alpha_2x_Up
STAT3high_ABC_DLBCL_subgroup
HRAS_overexpression_4x_up
Blood_Module−3.2_Inflammation−1
NFkB_Up_BCR_paper
Stromal−2_DLBCL_survival_predictor
Germinal_center_Boell_DLBCL
Macrophage_M0_Newman
Myeloma_MS_subgroup_up
pathway
−4
−3
−2
−1
0
NES

D

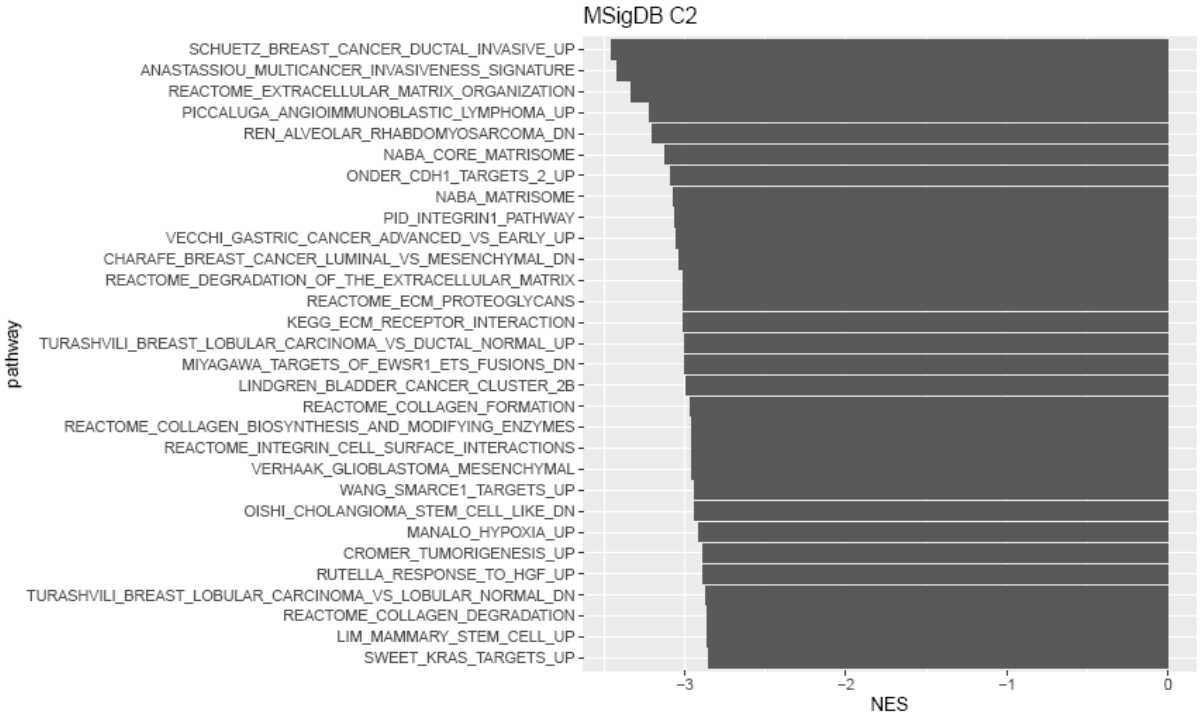
MSigDB C2
SCHUETZ_BREAST_CANCER_DUCTAL_INVASIVE_UP
ANASTASSIOU_MULTICANCER_INVASIVENESS_SIGNATURE
REACTOME_EXTRACELLULAR_MATRIX_ORGANIZATION
PICCALUGA_ANGIOIMMUNOBLASTIC_LYMPHOMA_UP
REN_ALVEOLAR_RHABDOMYOSARCOMA_DN
NABA_CORE_MATRISOME
ONDER_CDH1_TARGETS_2_UP
NABA_MATRISOME
PID_INTEGRIN1_PATHWAY
VECCHI_GASTRIC_CANCER_ADVANCED_VS_EARLY_UP
CHARAFE_BREAST_CANCER_LUMINAL_VS_MESENCHYMAL_DN
REACTOME_DEGRADATION_OF_THE_EXTRACELLULAR_MATRIX
REACTOME_ECM_PROTEOGLYCANS
KEGG_ECM_RECEPTOR_INTERACTION
TURASHVILI_BREAST_LOBULAR_CARCINOMA_VS_DUCTAL_NORMAL_UP
MIYAGAWA_TARGETS_OF_EWSR1_ETS_FUSIONS_DN
LINDGREN_BLADDER_CANCER_CLUSTER_2B
REACTOME_COLLAGEN_FORMATION
REACTOME_COLLAGEN_BIOSYNTHESIS_AND_MODIFYING_ENZYMES
REACTOME_INTEGRIN_CELL_SURFACE_INTERACTIONS
VERHAAK_GLIOBLASTOMA_MESENCHYMAL
WANG_SMARCE1_TARGETS_UP
OISHI_CHOLANGIOMA_STEM_CELL_LIKE_DN
MANALO_HYPOXIA_UP
CROMER_TUMORIGENESIS_UP
RUTELLA_RESPONSE_TO_HGF_UP
TURASHVILI_BREAST_LOBULAR_CARCINOMA_VS_LOBULAR_NORMAL_DN
REACTOME_COLLAGEN_DEGRADATION
LIM_MAMMARY_STEM_CELL_UP
SWEET_KRAS_TARGETS_UP
pathway
−3
−2
−1
0
NES

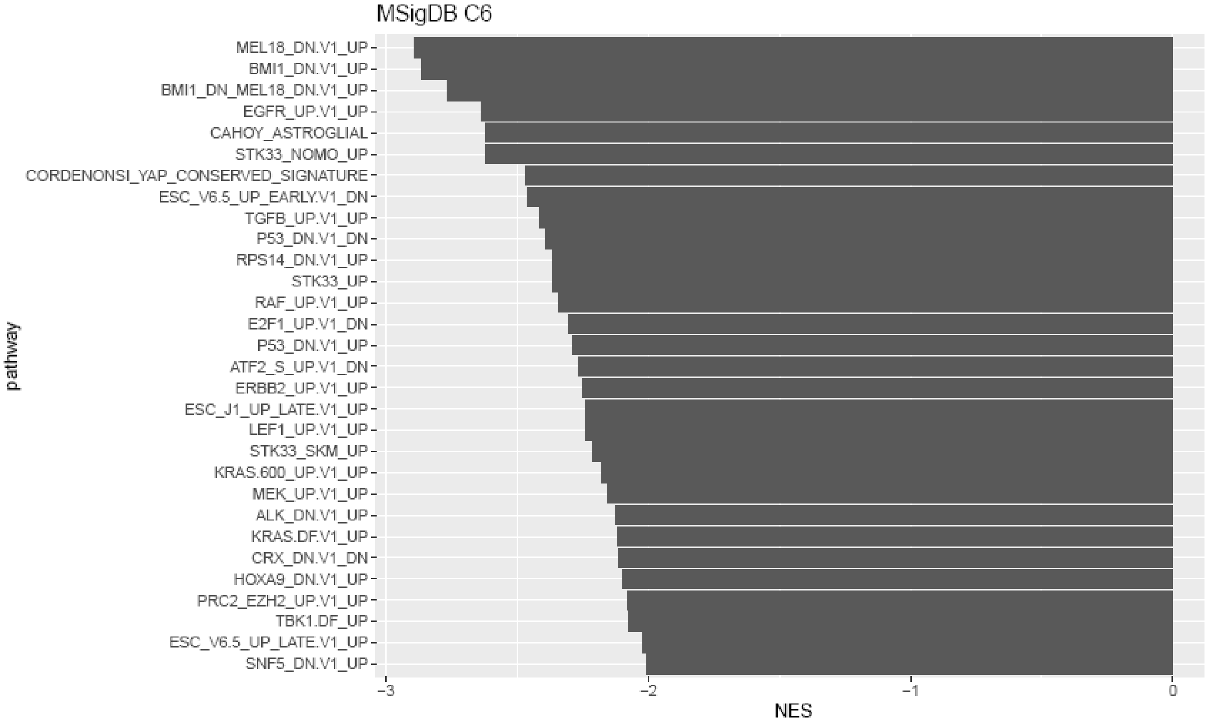
MSigDB C6
MEL18_DN.V1_UP
BMI1_DN.V1_UP
BMI1_DN_MEL18_DN.V1_UP
EGFR_UP.V1_UP
CAHOY_ASTROGLIAL
STK33_NOMO_UP
CORDENONSI_YAP_CONSERVED_SIGNATURE
ESC_V6.5_UP_EARLY.V1_DN
TGFB_UP.V1_UP
P53_DN.V1_DN
RPS14_DN.V1_UP
STK33_UP
RAF_UP.V1_UP
E2F1_UP.V1_DN
P53_DN.V1_UP
ATF2_S_UP.V1_DN
ERBB2_UP.V1_UP
ESC_J1_UP_LATE.V1_UP
LEF1_UP.V1_UP
STK33_SKM_UP
KRAS.600_UP.V1_UP
MEK_UP.V1_UP
ALK_DN.V1_UP
KRAS.DF.V1_UP
CRX_DN.V1_DN
HOXA9_DN.V1_UP
PRC2_EZH2_UP.V1_UP
TBK1.DF_UP
ESC_V6.5_UP_LATE.V1_UP
SNF5_DN.V1_UP
pathway
−3
−2
−1
0
NES

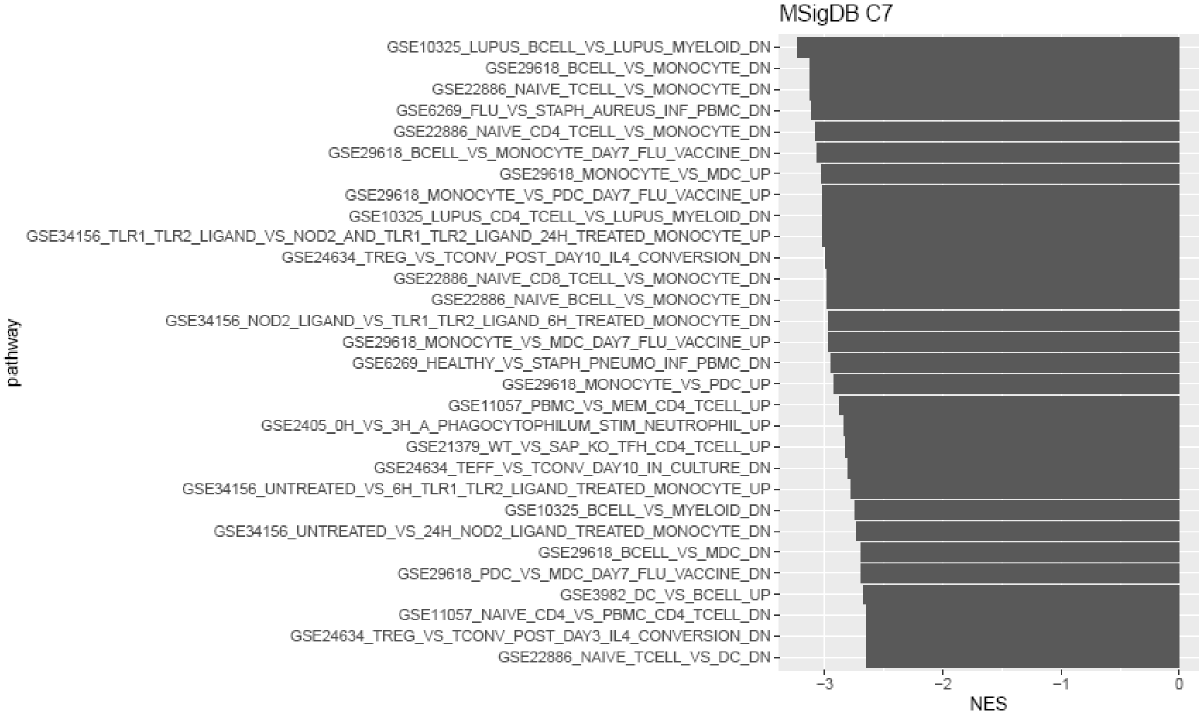

mSigDB, Molecular Signature Database; NES, normalized enrichment score; NMF, non-negative matrix factorization

References

1. Wright G, Tan B, Rosenwald A, Hurt EH, Wiestner A, Staudt LM. A gene expression-based method to diagnose clinically distinct subgroups of diffuse large B cell lymphoma. *Proc Natl Acad Sci U S A*. 2003;100(17):9991-9996.

2. Pfeifer M, Grau M, Lenze D, et al. PTEN loss defines a PI3K/AKT pathway-dependent germinal center subtype of diffuse large B-cell lymphoma. *Proc Natl Acad Sci U S A*. 2013;110(30):12420-12425.

3. Lenz G, Wright G, Dave SS, et al. Stromal gene signatures in large-B-cell lymphomas. *N Engl J Med*. 2008;359(22):2313-2323.

4. Shaffer AL, Wright G, Yang L, et al. A library of gene expression signatures to illuminate normal and pathological lymphoid biology. *Immunol Rev*. 2006;210:67-85.
